# DNA origami–enhanced force spectroscopy and AlphaFold structural analyses reveal the folding landscape of calcium-binding proteins

**DOI:** 10.1126/sciadv.adv1962

**Published:** 2025-04-30

**Authors:** Honglu Zhang, Cristhian Cañari-Chumpitaz, Lisa Alexander, Huan Zhang, Chunhai Fan, Carlos Bustamante

**Affiliations:** ^1^ School of Sensing Science and Engineering, School of Electronic Information and Electrical Engineering, Shanghai Jiao Tong University, Shanghai 200240, China.; ^2^ Jason L. Choy Laboratory for Single Molecule Biophysics. Institute for Quantitative Biosciences-QB3, University of California, Berkeley, Berkeley, CA 94720, USA.; ^3^ Department of Chemistry, University of California, Berkeley, Berkeley, CA 94720, USA.; ^4^ Howard Hughes Medical Institute, Stanford University, Stanford, CA 94305, USA.; ^5^ Metagenomi Inc., Discovery, Emeryville, CA 94608, USA.; ^6^ School of Agriculture and Biology, Shanghai Jiao Tong University, Shanghai 200240, China.; ^7^ School of Chemistry and Chemical Engineering, Frontiers Science Center for Transformative Molecules, National Center for Translational Medicine, Shanghai Jiao Tong University, Shanghai 200240, China.; ^8^ Shanghai Key Laboratory for Nucleic Acids Chemistry and Nanomedicine, Institute of Molecular Medicine, Renji Hospital, School of Medicine, Shanghai Jiao Tong University, Shanghai 200240, China.; ^9^ Department of Molecular and Cell Biology, University of California, Berkeley, Berkeley, CA 94720, USA.; ^10^ Department of Physics, University of California, Berkeley, Berkeley, CA 94720, USA.; ^11^ Howard Hughes Medical Institute, University of California, Berkeley, Berkeley, CA 94720, USA.; ^12^ Kavli Energy Nanoscience Institute, University of California, Berkeley, Berkeley, CA 94720, USA.

## Abstract

Understanding the intricate folding process of proteins and characterizing the intermediates they populate en route to their native state remain challenging despite the remarkable accuracy achieved through in silico approaches for predicting native protein structures. Here, we replaced the conventional flexible double-stranded DNA handle force transducers with solid DNA-origami bundles to conduct single-molecule folding force-spectroscopy studies on calerythrin, a compact multidomain calcium-binding globular protein. The resulting origami-enhanced data revealed a previously “hidden” folding intermediate and the hierarchical nature of the protein’s folding pathway. A systematic comparison of the AlphaFold-predicted conformational ensemble of structures of the native state and folding intermediates across various calcium-binding proteins provides a structural rationalization for the folding behavior of this protein family. The integration of DNA origami–enhanced single-molecule experiments with in silico approaches, and structural analysis presented here, constitutes a comprehensive method to uncover the rules underlying the formation of intermediates within protein folding landscapes.

## Teaser

DNA origami force probes and AlphaFold combine experiments and AI to decode the dynamics of protein folding.

## INTRODUCTION

Understanding how a protein’s amino acid sequence determines its folding pathway remains an active area of research (*1*–*4*). Moreover, conditions leading to protein misfolding have become a subject of increasing inquiry since these processes are associated with several debilitating illnesses, such as Alzheimer’s disease, Parkinson’s, and type II diabetes (*5*). Recently, AlphaFold (*6*, *7*), a deep learning–based approach achieved remarkable accuracy in predicting native protein structures. While the accurate prediction of the folded structure is undoubtedly valuable in advancing our understanding of protein functionality, the resulting static structure does not provide insight into the intricacies of the folding process itself. Thus, investigating the hierarchical nature of the folding pathways, the intermediate states during the folding process, and the kinetic factors that bring about native or misfolded states, is essential to comprehensively understand protein folding.

**Fig. 1.**
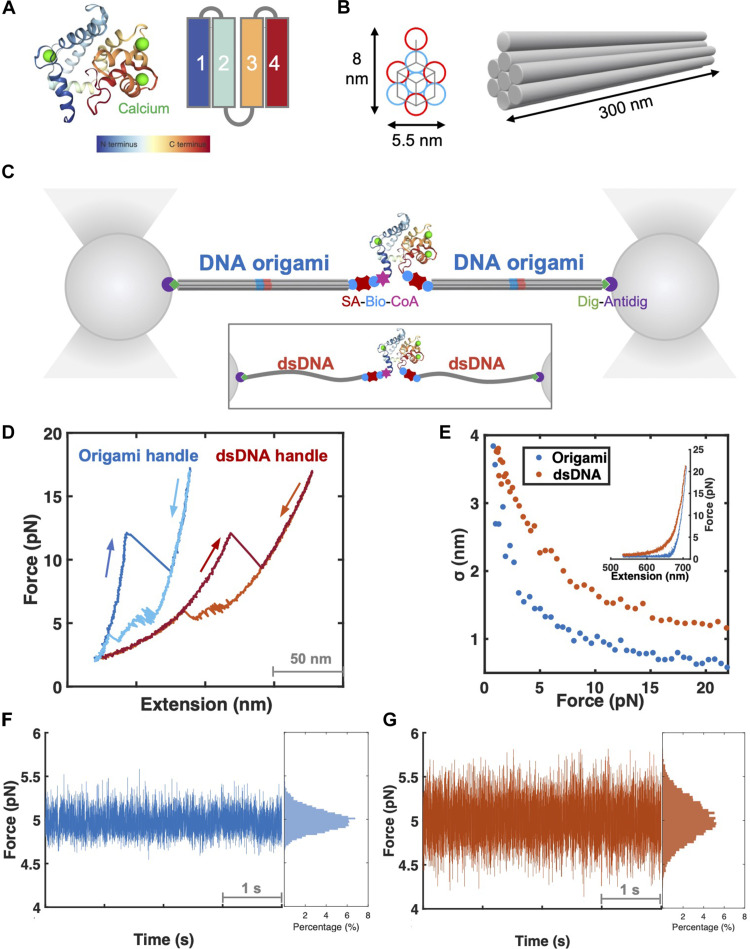
Force-extension curves of CaE with DNA origami or dsDNA handles. (**A**) Schematic illustration of CaE (PDB: 1NYA). Blue color depicts the N terminus and red color the C terminus. (**B**) Schematic illustration of single DNA origami of eight-helix hexagonal lattice. Each gray pillar represents a bundle of DNA double helix. The bundles are color-coded to indicate the polarity (5′end to 3′ end) of the scaffold strand. The scaffold strand threads through DNA double helices coded in blue color for one polarity and through their counterparts coded in red with opposite polarities. Staple strand-crossover (gray color) patterns of cross-sectional slices. (**C**) Geometry of optical tweezers experiment with DNA origami or dsDNA (inset) handles. (**D**) Representative force-extension curves of tweezing CaE with DNA origami (blue) or dsDNA (red) handles. Force-dependent noise amplitudes [(**E**) root mean square displacement of trapped beads, σ], representative force-extension curves [(E) inset], and force-time traces of tweezing with DNA origami [(**F**) blue] and dsDNA [(**G**) red] handles.

Calcium-binding proteins, essential for cell signaling through Ca^2+^-dependent intra- or interdomain rearrangements, display diverse but poorly understood folding mechanisms, with research limited to a few family members (*8*–*13*). To investigate these mechanisms, we analyzed calerythrin [(CaE) Protein Data Bank (PDB): 1NYA], a 20-kDa protein from *Saccharopolyspora erythraea*, which functions as a calcium buffer to maintain stable free Ca^2+^ concentrations in prokaryotic cells (*14*–*16*). CaE features a compact structure with four EF hands (helix-loop-helix) motifs within its N- and C-terminal globular domains, three of which bind calcium. Notably, EF-hand motif 2 (EF2) serves as an atypical nonbinding site, contributing to its unique functional properties (Fig. 1, fig. S1, and tables S1 and S2).

Unlike ensemble kinetic studies that average out the detailed dynamics of protein folding, single-molecule methods allow real-time tracking of the folding and unfolding trajectories of individual proteins, including the sequence of folding intermediates (*17*–*19*). However, the limited spatiotemporal resolution of current single-molecule force spectroscopy instruments and the transient nature of folding intermediates often make it difficult to discern the complete set of intermediates within a protein’s folding pathway. Accordingly, efforts have been made to enhance the resolution of single-molecule force spectroscopy assays using optical tweezers. Typically, these assays involve trapping plastic or glass beads ~300 times larger than proteins. Consequently, “molecular handles” of double-stranded DNA (dsDNA) segments (*20*) are used to connect the beads and the protein. It can be shown (*21*) that the signal-to-noise ratio of the measurement is given by

$$\mathrm{SNR}=\frac{\kappa_{\text{molecular}}}{2\sqrt{k_{B}T\gamma B}}\mathrm{\Delta x}$$ (1)

where Δx is the change in extension of the polypeptide upon a given folding transition, *k_B_* is the Boltzmann’s constant, *T* is the absolute temperature, γ is the drag coefficient of the beads in the aqueous medium, and *B* is the bandwidth of the measurement. Here, κ*_molecular_* represents the effective stiffness of the experiment in terms of those of the handles, κ*_handles_*, and of the tethered molecule, κ*_polypeptide_* (*21*)

$$\kappa_{\text{molecular}}=\frac{1}{\frac{1}{\kappa_{\text{handles}}}+\frac{1}{\kappa_{\text{polypeptide}}}}$$ (2)

thus, the molecular stiffness attains a maximum value, equal to κ*_polypeptide_* when the handles are infinitely stiff. Since the stiffness is inversely proportional to their length, shorter handles have been tried (*22*), but they have the disadvantage that the molecule can be damaged by the closer proximity of the trapping lasers. An alternative approach is to replace the flexible DNA handles with more rigid DNA origami transducers (*23*, *24*).

Here, we report that the use of DNA-origami force transducers not only improves the spatiotemporal resolution of optical tweezers measurements but also allowed us to uncover a “hidden” intermediate in the folding landscape of CaE, an intermediate undetected with conventional dsDNA handles. We also used AF-Cluster (*25*), an improved deep-learning framework of AlphaFold, to predict the diverse conformations attained by both the native and folding intermediates of CaE and those of two related calcium-binding protein subfamilies, observed via optical tweezers. The combined approach substantially advances our understanding of protein folding dynamics and enhances our understanding on protein structure studies.

## RESULTS

### Comparison of optical tweezers experiments using DNA origami and dsDNA handles

To elucidate the performance of DNA-origami transducers, we compared the force-extension behavior of individual CaE molecules tethered with DNA-origami and dsDNA handles of the same length (Fig. 1). The DNA-origami architecture was designed as a bundle of eight parallel DNA duplexes arranged in a hexagonal lattice pattern (Fig. 1B, figs. S2 to S4, and table S3) (*26*). The N and C termini of CaE were modified with biotin molecules to attach the protein to handles (DNA-origami or dsDNA) labeled with streptavidin at one end. The other end of the handle was attached to 1-μm-sized beads via digoxigenin-antibody linkages (Fig. 1C, fig. S5, and table S4). Originally, we designed the eight-helix origami with a contour length of 300 nm. However, to prevent potential photodamage to the protein by the trapping beams, dimeric origami handles approximately 600 nm long were used on both sides of the protein (fig. S5 and table S4). Accordingly, 1800–base pair dsDNA handles with an equivalent contour length were used for comparison.

Individual CaE molecules were subjected to stretching and relaxing force ramp cycles at a constant rate in an optical tweezers instrument, using either origami or dsDNA handles (Fig. 1D and fig. S6). Characteristic cooperative unfolding and refolding transitions (rips and zips, respectively) can be seen with both types of transducers. The magnitude of these transitions and the forces at which they appear are consistent with previous values observed with this protein (*19*). However, the elastic response of the two transducers is notably different, reflecting their distinct mechanical stiffnesses (Fig. 1E, inset). The noise amplitude is significantly smaller with the origami handles, especially in the low-force regime (<10 pN); at pulling forces of ~5 pN, the noise is suppressed by about 46% with the origami handles compared to that of DNA (Fig. 1, E to G, and fig. S7).

### Nonequilibrium unfolding transition analysis

**Fig. 2.**
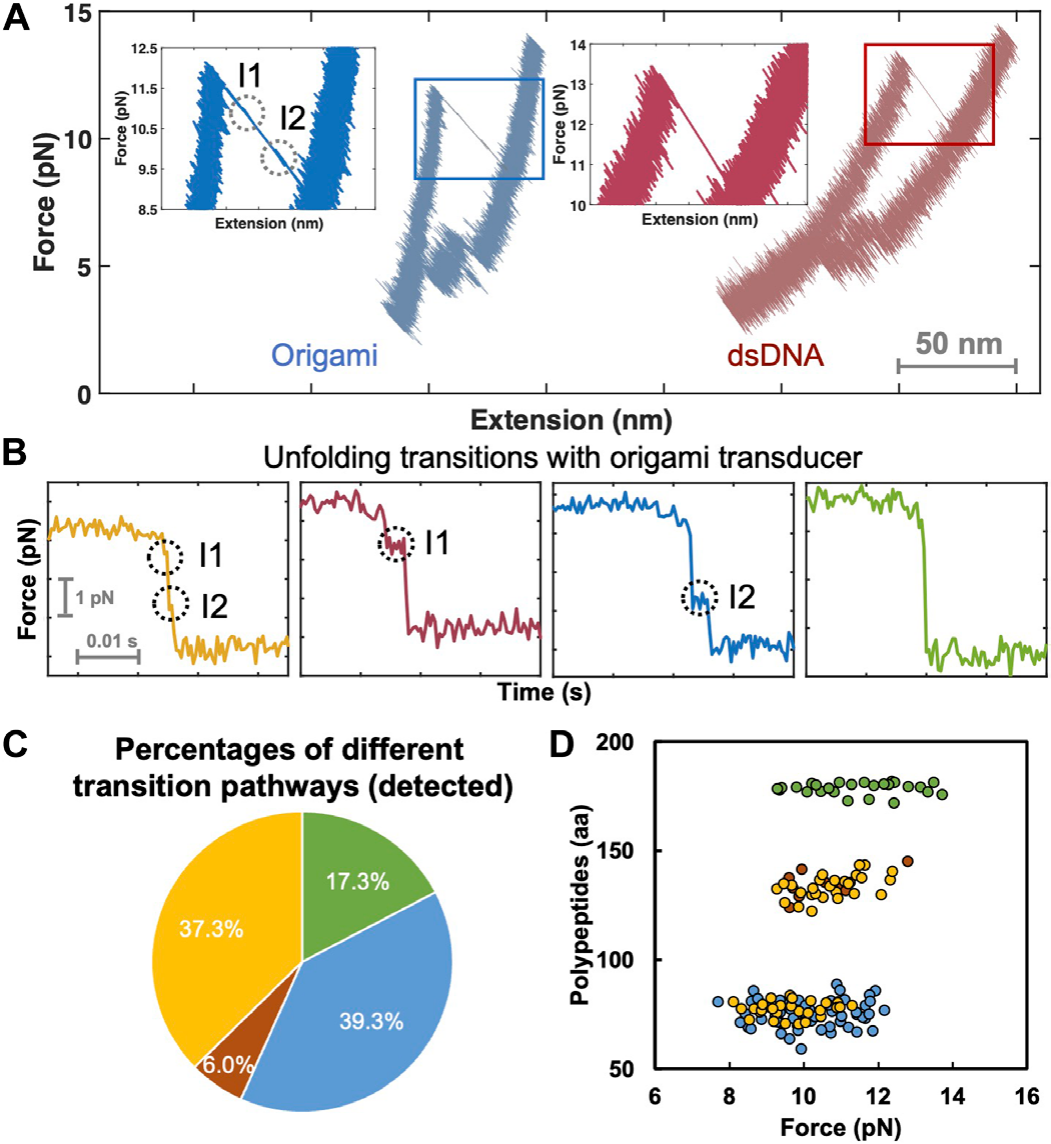
Nonequilibrium unfolding transitions. (**A**) Representative force-extension curves using DNA origami and dsDNA handles. (**B**) Representative high-resolution force versus time trajectories using the DNA origami transducer during the unfolding of CaE. The panels depict trajectories indicating the presence of either two intermediates (yellow curve), one intermediate (red and blue curves), or no intermediates (green curve). The trajectories obtained with dsDNA do not reveal intermediates. Chart indicating the percentage of different unfolding transition pathways (**C**) and contour length changes in the numbers of amino acids (aa) corresponding to the unfolding transitions (**D**) observed in (B). Representative 150 events from 32 molecules were analyzed using the same color code as in (B).

To investigate the force-extension response and force-dependent transitions of CaE at the single-molecule level, we conducted a detailed analysis of data collected with both origami and dsDNA handles (Fig. 2). With dsDNA handles, force ramp experiments show that CaE unfolds in a cooperative two-state process and refolds, populating a refolding intermediate (Fig. 2A, right). At the pulling rates used (100 nm/s), CaE unfolds at 13 ± 1.5 pN (SD) in a single rip and refolds in two steps, consistent with previous studies (*19*). In contrast, the force-extension curves of CaE with the origami transducer, at the same pulling rate (Fig. 2A, left), reveal two unfolding intermediates labeled I1 and I2. As shown in Fig. 2B, these two unfolding intermediates are populated transiently and stochastically; they are too short-lived to be captured consistently in the trajectories obtained with origami handles and are not detected with conventional dsDNA handles. Figure 2C shows the distribution of cases observing two (yellow), one (red or blue), or no (green) intermediates in successive unfolding transitions of CaE force-extension curves using origami handles. Notably, about 37.3% of the traces reveal both intermediates during the unfolding experiments with the stiffer handles.

Next, we used the end-to-end distances associated with the observed intermediates to calculate the contour lengths involved in these unfolding transitions using the worm-like-chain model of polymer elasticity (Fig. 2D) (*27*). The full unzipping transition from the fully folded (F) to the unfolded (U) state is 65 ± 3 nm, equivalent to 177 ± 6 amino acids, consistent with the full length of CaE. The polypeptide length associated with I1 was calculated to be 43 ± 3 amino acids, representing a smaller unfolding transition than that of the N or C domains. The polypeptide length for the transition from I1 to I2 was 49 ± 3 amino acids. Together, these two transitions add up to 92 ± 5 amino acids, very close to the contour length of the N domain (1 to 90, 90 amino acids in length). The contour length associated with the transition from I2 to the fully unfolded protein was 81 ± 6 amino acids, close to the contour length of the C domain (91 to 177, 87 amino acids in length). To better determine which parts of the protein are involved in the various unfolding and refolding events, we conducted experiments in passive mode (constant trap position).

### Equilibrium transition analysis with passive mode experiments

**Fig. 3.**
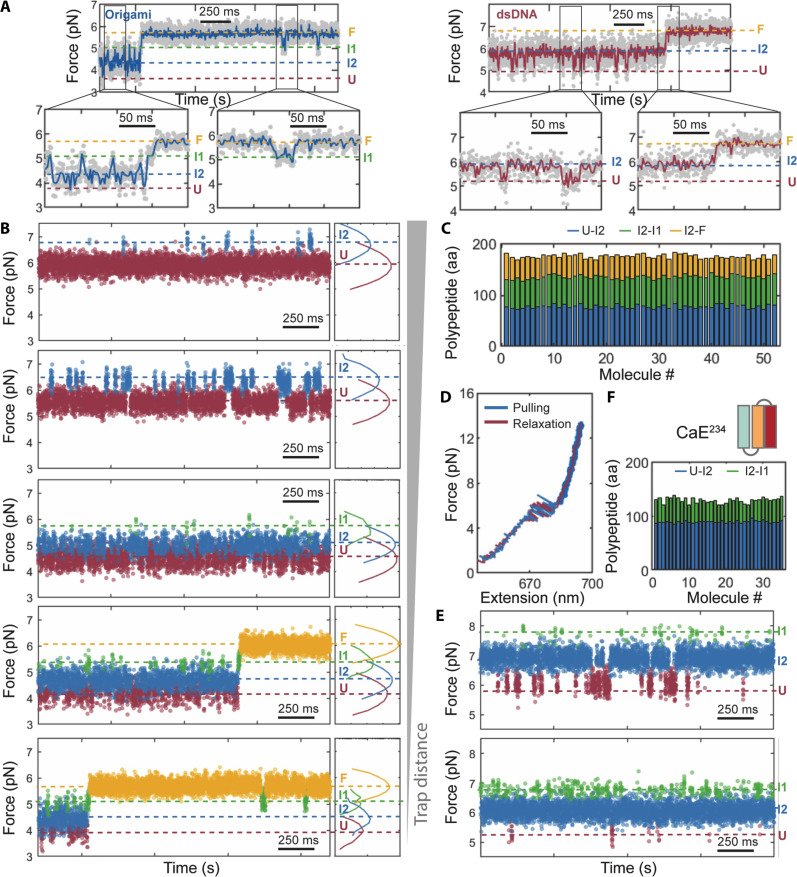
Equilibrium transitions with passive mode experiments. (**A**) Representative 2-s-long traces displaying the force fluctuations associated with folding and unfolding transitions of a single CaE molecule obtained with origami (left) or with dsDNA (right) handles using passive mode. Insets, transitions observed in expanded 300- or 500-ms windows. (**B**) Representative force-versus-time traces of CaE at different preset distances. In the high force regime (corresponds to large trap distance, uppermost traces), only transition between U and I (I1 and/or I2) could be observed with the origami handles. With decreased trap distances, it is possible to observe more transitions (I to I1, I1 to F, and I2 to F). (**C**) Contour lengths associated with different states relative to the folded state in passive mode with origami handles for 51 molecules. (**D**) Representative force-extension curves of CaE^234^ with origami handles. (**E**) Representative passive mode traces during 1 s of a single CaE^234^ molecule obtained at a constant trap distance using origami handles. (**F**) Contour lengths associated with different states relative to the folded state in passive mode with origami handles for 35 molecules.

Equilibrium folding/unfolding experiments were carried out in passive mode in which the protein is stretched to a preset and fixed trap distance and whose transitions between folded and unfolded states are reflected in force changes in real time (Fig. 3A). This mode allows for the monitoring of fast transitions without the time-resolution limitations imposed by the force-feedback system used in constant force experiments (*28*). Representative passive mode traces of CaE, obtained with origami and dsDNA handles, are shown in Fig. 3A (left and right, respectively). Force fluctuations are noticeably smaller with origami handles. Expanded views covering 200 ms (insets) reveal four different populated folding states with origami handles, whereas only three states are observed with dsDNA handles (right panel and insets). Bayesian hidden Markov model analysis confirmed the existence of at least four distinct states in the origami data, labeled F, U, intermediates I1, and I2. Note that I1 is not detected with dsDNA handles.

By tuning the preset trap distance in passive mode, we can tilt the equilibrium toward the folded or unfolded state and dissect the population of states and the transition paths within the CaE folding landscape. Five representative time traces obtained at different trap distances in this mode are shown in Fig. 3B. A distinct shift in population from the unfolded state to the fully folded state is observed as the trap distance decreases (lowering the applied mean force). Initially, the fully unfolded state predominates at a high trap distance (Fig. 3B, top). However, as this distance is reduced, folded intermediates (I2 and then I1) become increasingly populated until the fully folded protein becomes the dominant form (Fig. 3B, bottom). Notably, transitions between the intermediates I1 and I2, as well as between the I1 and the fully folded state, could be detected using origami transducers despite the short-lived nature of the I1 at all trap distances. The force-dependent unfolding and refolding transition rates of CaE are determined by measuring the distributions of lifetimes in passive mode experiments (fig. S8A).

Figure 3C depicts the contour lengths associated with all three states relative to the folded state, as obtained in passive mode with origami handles. The contour length of the folded state is 178 ± 3 amino acids, consistent with the expected value of 177 amino acids. The contour length changes associated with the U to I1 and U to I2 transitions are 137 ± 5 amino acids and 85 ± 4 amino acids, respectively, consistent with the refolding of three EF hands (EF234, expected value 133 amino acids) and EF34 (C domain, expected value 87 amino acids). The contour length associated with the U to I2 transition, observed here in passive mode corresponding to the folding of the C domain of CaE, aligns well with the value obtained in previous passive mode experiments performed with dsDNA handles (*26*) and with the values from force-ramp experiments (Fig. 2D). Similarly, the contour length determined for the U to I1 transition coincides with that obtained in force-ramp experiments (Fig. 2D).

To further verify the structural assignments underlying distinct states, protein variant CaE^234^ with EF1 hand deletion was tested in single-molecule force-ramp experiments using DNA-origami force transducers. Notably, CaE^234^ displayed a short-lived state involving all three EF hands, identical to the intermediate I1 (Fig. 3, D and E), and transitioned to a shorter intermediate. Kinetic analysis and the measured length (~87 ± 4 amino acids) of this intermediate support its identification with the wild-type’s C domain (EF34) (figs. S8B and S3F). This analysis confirmed that I1 represents the folding of three EF hands, which occurs only when EF2 can interact with and fold onto the fully prefolded C domain, suggesting domain folding cooperativity. This pattern mirrors calmodulin (CaM, PDB: 1CLL), where EF3 rarely folds without the N domain being prefolded (*11*).

### The folding pattern of CaE

**Fig. 4.**
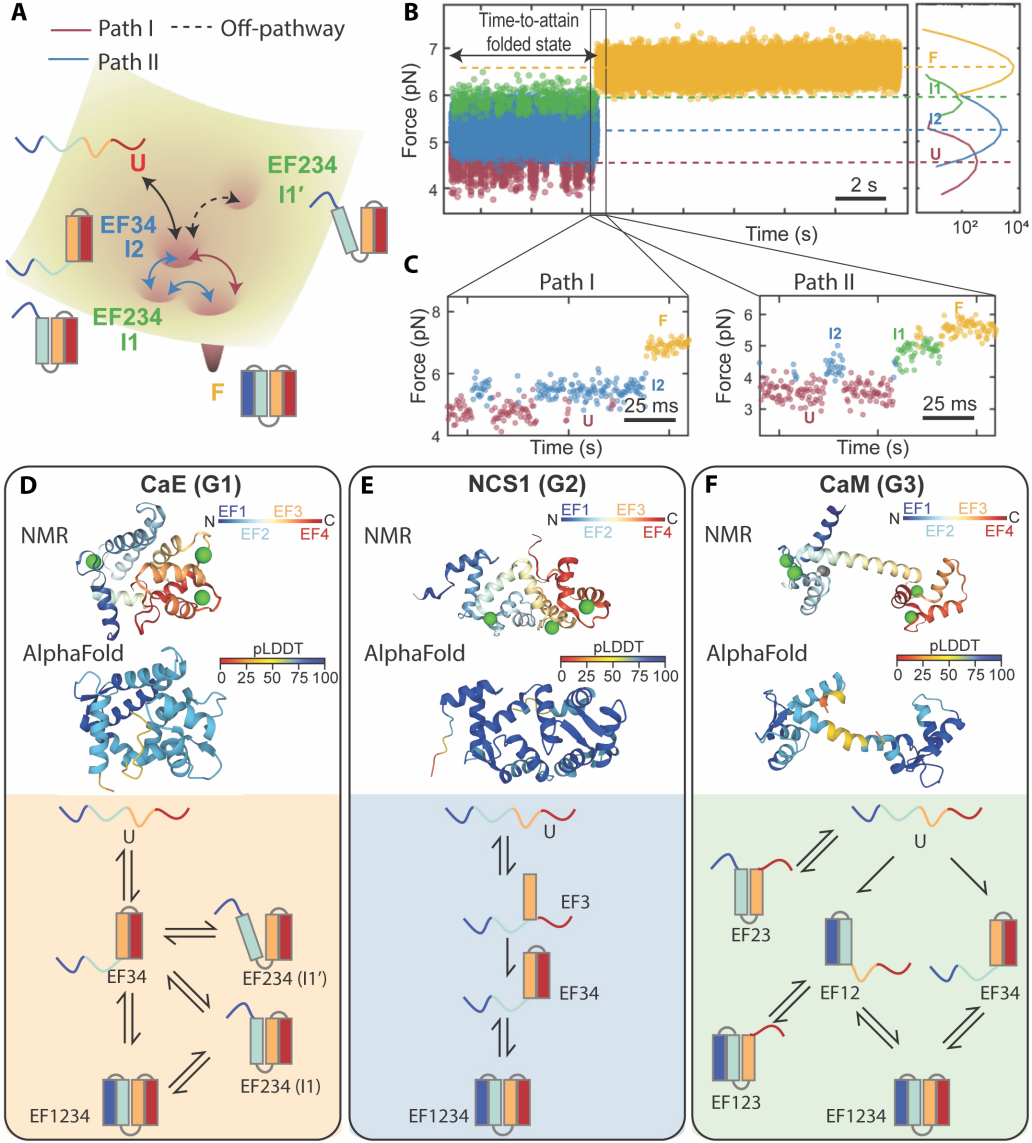
Folding landscapes and structures of calcium-binding proteins. (**A**) Folding landscape of CaE with transition paths among various states. (**B** and **C**) Representative force-versus-time traces of CaE showing two transition paths (I and II). Path I, U-I2-F; path II, U-I2-I1-F. (**D** to **F**) Comparison on the structures generated with NMR, models predicted with AlphaFold, and folding patterns of three proteins representing each subfamily of calcium-binding proteins. Left, CaE of group 1 (G1) contains two domains that reside on the opposite face of a short linker. Middle, NCS1 of group 2 (G2) contains two domains that reside on the same face of a short linker. Right, CaM of group 3 (G3) contains two globular domains separating by a flexible linker. The prediction of AlphaFold models is evaluated with a per-residue confidence score (pLDDT, predicted local distance difference test; between 0 and 100, with 100 being the best).

On the basis of the above observations, the folding landscape of CaE, including all possible transition paths among various states, was reconstructed (Fig. 4A). During calcium-dependent folding, the last four α helices (EF3 and EF4) fold cooperatively, forming the folded C domain (I2). Then, EF1 and EF2 can cooperatively fold onto the prefolded I2 to achieve the fully folded protein (path I in Fig. 4, A to C). Alternatively, EF2 may fold first onto the prefolded I2 to form EF234. The resulting intermediate (EF234) does not always appear on the direct pathway to the fully folded state. Instead, EF234 frequently interconverts with I2 for a notable period before CaE attains its fully folded conformation (Fig. 4, B and C). We speculate that this interconversion involves the cis-trans isomerization of Pro^103^ in the linker between EF2 and EF3 (fig. S9), which in turn influences the folding pathways. When Pro^103^ is in cis conformation, it results in the off-pathway intermediate I1′, blocking complete folding. In contrast, when Pro^103^ adopts the trans conformation, it enables the formation of I1, allowing EF1 to fold and achieve the fully native CaE structure (path II in Fig. 4, A and B).

### Comparison of the folding patterns in calcium-binding protein families

To gain more insight on the factors that control the folding of calcium-binding proteins, we compared the folding characteristics of CaE to those of other EF-hand proteins such as CaM and the neuronal calcium sensor 1(NCS-1, PDB: 2LCP) using experimentally solved nuclear magnetic resonance (NMR) structures and AlphaFold predictions (*6*, *29*). These three proteins represent three distinct groups of EF-hand calcium-binding proteins, respectively. They exhibit both unique and shared folding mechanisms, as revealed through single-molecule force spectroscopy. As shown in Fig. 4 (D to F), NMR and AlphaFold analysis of the full-length proteins shows that while most EF-hand proteins are structured into two domains, each comprising a pair of EF-hands, they can be divided into three groups based on domain arrangements and structural plasticity. Group 1 including CaE and invertebrate sarcoplasmic calcium–binding proteins (SCPs), exhibits a compact globular structure with two pairs of EF-hands connected by a short and tight linker (Fig. 4D). Group 2 consisting of proteins like recoverin and other members of the NCS subfamily, has a kinked helix that positions EF-hands in a tandem layout on one side of the protein (Fig. 4E). Group 3, which includes CaM and related proteins, features two globular domains connected by a flexible linker that facilitates diverse orientations and interactions between them (Fig. 4F).

A comparison analysis of the folding patterns obtained through single-molecule force spectroscopy studies across these subfamilies highlights both unique and shared folding mechanisms within the EF-hand protein family. Rief *et al.* (*11*) found that CaM’s EF-hand pairs could fold independently without a preferred folding order (Fig. 4F, bottom). Conversely, Cecconi *et al.* (*12*) observed that NCS1 folds in a strict order starting with EF3, followed by EF4, and then EF12 (Fig. 4E, bottom). Our studies on CaE, despite its low sequence homology to NCS1, also reveal a rigorous folding order where the calcium-binding C domain (EF34) folds first, leading to different scenarios: either EF2 folds next followed by EF1, or EF1 and EF2 fold concurrently (Fig. 4D, bottom).

**Fig. 5.**
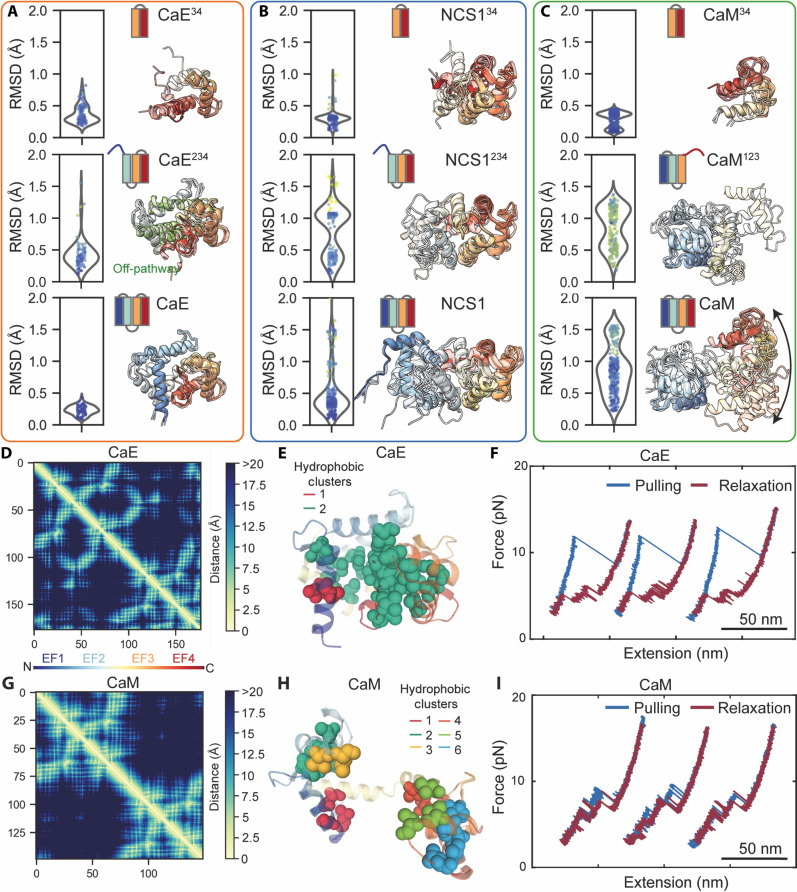
Conformational distributions and intramolecular interactions analysis of CaM, NCS1, and CaE proteins. An ensemble of conformational states was predicted using AF-Cluster for CaE (**A**), NCS1 (**B**), and CaM (**C**), including their truncated proteins. A superimposition of conformational states for each protein is shown, along with a violin plot of the root mean square displacement (RMSD) with respect to the corresponding AlphaFold prediction. Conformational heterogeneity is observed as a spread in the RMSD and different clusters using principal components analysis (fig. S10). A comparison of contact maps, hydrophobic cluster analysis, and force-extension curves of CaE (**D** to **F**) and CaM (**G** to **I**) was performed using DNA origami transducers.

Next, we used AF-Cluster to analyze the conformational space that such intermediates explore by clustering multiple-sequence alignments (*25*). The folded C-terminal domain (EF34) of these proteins exhibited predicted structures that were clustered from their respective AlphaFold models and were in close agreement with the C domain of their full-domain proteins (Fig. 5 and fig. S10). This finding aligns with observations that the C domain provides a stable scaffold for the folding of CaE, NCS1, and CaM. When comparing intermediates with three EF-hands (EF234), we noticed considerable deviations from the native folding of each respective protein. In the cases of NCS1 and CaM, these deviations were mostly explained by the flexible nature of the linker between EF2 and EF3 hands (Fig. 5, B and C). For CaE, the EF234 (CaE^234^) predictions clustered very close to the native folding, although alternative structures mainly differing in the conformation of the EF2 hand were predicted. We hypothesize that this structure could be related to the intermediate I1′, a misfolded state that does not allow the folding of EF1 due to steric clashes (Fig. 5A). Last, an analysis of full-length proteins revealed notable differences between NCS1 and CaM compared to CaE. The predicted structures for CaE showed small deviations from each other, consistent with the compact nature of this protein. On the other hand, NCS1 and CaM exhibited a wide range of conformations due to increased degrees of freedom allowed by the lengths of their linkers (Fig. 5, B and C). AF-Cluster was able to predict a wide range of conformations for CaM, which have been observed experimentally by NMR.

It is possible to rationalize the distinct folding behaviors of CaE, NCS1, and CaM based on their structural conformations, calcium-binding capabilities, and interaction dynamics. CaM’s flexible α helix linker and its two pairs of EF-hands capable of binding calcium permits these pairs to have autonomous stability and to fold independently in any folding order. In contrast, CaE featuring shorter, stiffer linkers that restrict domain orientation promotes a more compact structure and reduces the folding independence of the N domain. Notably, in CaE and NCS1, one EF hand (EF2 in CaE, EF1 in NCS1) does not bind calcium, which limits folding autonomy and leads to sequential folding onto a preformed C domain. These factors—linker length and flexibility, along with calcium-induced stability—may contribute to the varying folding patterns and functional diversity among these proteins.

### Intramolecular interactions of calcium-binding proteins

Proteins undergo a complex process of folding, which is facilitated by a dynamic network of noncovalent interactions, including hydrogen bonds, Van der Waals forces, as well as hydrophobic and ionic interactions. To gain insight into the intramolecular interactions underlying the mechanical stability of these calcium-binding proteins, we used ProteinTools, a toolkit to identify hydrophobic clusters, hydrogen bond networks, salt bridges, and contact maps. This analysis was based on the structural data from NMR and AlphaFold for CaE, NCS1, and CaM (Fig. 5, figs. S11 and S12, and tables S5 to S10).

An analysis of the contact maps for these proteins reveals that CaE is not only more compact, but all four of its EF hands have 177 amino acids residues spaced within 5 Å, as seen as off-diagonal peaks in the contact map, particularly between EF1 and EF4 (Fig. 5D and fig. S11). These conclusions are also supported by a large hydrophobic cluster observed within the CaE structure (Fig. 5E and figs. S11 and S12), in contrast to the nearly four-fold smaller clusters found in NCS1 and CaM (Fig. 5H and figs. S11 and S12). Conversely, NCS1 exhibits a lower degree of compaction, with contacts between EF1 and EF4 absent (fig. S11); similarly, the hydrophobic clusters in these two proteins are spread over different regions of the proteins (figs. S11 and S12), providing stability to each EF hand independently but not to the entire structure as seen in CaE. Last, CaM exhibits contacts only connecting EF1-EF2 and EF3-EF4 (Fig. 5G and fig. S11), but the long and flexible linker between each domain allows for independent folding of the N domain and C domain, as observed in force-spectroscopy studies (Fig. 5I). Overall, this structural analysis also explains the low mechanical stability of CaM, which, when pulled with a DNA-origami force transducer, exhibits unfolding transitions at forces as low as ~4 pN, corresponding to the rupture of the EF1, EF12, or EF34 hands independently. On the other hand, CaE exhibits higher mechanical stability, with a rupture force of ~12 pN and high hysteresis between folding and unfolding, as its unfolding requires rupture of contacts that stabilize the overall 3D conformation. Only upon a major rupture the structure breaks into EF234 and EF34, transitions that are only discernable with the use of DNA-origami force transducers (Fig. 5F).

## DISCUSSION

We have used rigid DNA origami handles to uncover a hidden intermediate in the mechanical unfolding of CaE, obtaining a more complete picture of the protein’s hierarchical folding pathway and its interdomain cooperativity. We have combined this newly acquired information with deep-learning AlphaFold predictions and structural information to rationalize the difference between CaE’s folding landscape and those of other EF-hand proteins such as CaM and the neuronal calcium–binding protein subfamilies. This multi-prong analysis constitutes a powerful way to explore protein folding rules and yields a more comprehensive picture of protein folding landscapes.

Our experimental approach leverages the precision and versatility of DNA origami to study protein folding with enhanced resolution. This technique is adaptable to study a wide range of proteins, including larger or highly flexible ones, as well as other functional biomolecules such as ribozymes and long noncoding RNAs. In particular, our approach could facilitate the study of nascent polypeptide chain folding during real-time translation—a process that has traditionally been challenging to investigate.

Despite these advancements, certain challenges persist. Studying heterogeneous proteins or membrane-associated proteins remains difficult, as these often require specialized handling to ensure proper folding and integration into membranes. In addition, further improvements in resolution are necessary to eliminate additional sources of noise over the Brownian noise floor. Future efforts will focus on integrating our system with other approaches that enhance spatiotemporal resolution. For example, using smaller probes made from high-refractive index materials (*30*) or incorporating upconverting nanoparticles (*31*) could further refine the precision of force measurements.

While artificial intelligence (AI) predictions are advancing, accurately forecasting the dynamic process of protein folding in real-time remains an unsolved challenge. Our experimental setup offers valuable insights into folding pathways, but real-time predictions of the dynamic folding process would further enhance our understanding of how proteins navigate their folding landscapes. Advancements in AI and computational power may eventually enable more precise predictions of these dynamic processes, leading to more accurate experimental designs.

## MATERIALS AND METHODS

### Sample preparation

Each handle of DNA-origami transducers is designed and constructed by end-to-end connection of two eight-DNA helices packed on hexagonal lattice. The DNA-origami structures were designed using Cadnano2 software (*32*) and prepared by annealing the single scaffold stranded M13mp18 (New England BioLabs, 100 nM) with staple strands (synthesized at Integrated DNA Technologies, each at 200 nM) in 1× TAE-Mg^2+^ buffer [40 mM tris, 20 mM acetic acid, 2 mM EDTA, and 12.5 mM magnesium acetate (pH 8.0)] with a ratio of 1:10 from 95°C to room temperature in a rate of 1°C min^−1^. The sequences of staple strands are listed on table S1. Each origami handle is assembled with corresponding biotin- or digoxigenin-labeled staple strands and overhangs at the two distal ends. Multiple attachment points provide additional mechanical stability when the tether is under tension in tweezering. Afterward, all folded DNA origami bundles were purified by agarose gel electrophoresis (containing 1% agarose, Bio-Rad). Gel and running buffer contained 1× TAE-Mg^2+^ buffer and ran at 100 V for 1.5 hours at the cold room. The gels were visualized under ultraviolet light, and leading bands containing the origami bundles were cut and subsequently extracted using Freeze-N-Squeeze DNA Gel Extraction Spin Columns (Bio-Rad) in a centrifuge at 10,000 rcf for 8 min. Biotin-/digoxigenin-labeled origami monomers were dimerized by mixing them in a ratio of 1:1 and then incubated at constant temperatures of 37°C. The finial formed dimeric origami handles were stored at 4°C in the 1× TAE-Mg^2+^ buffer and used for further optical tweezering experiments. dsDNA handles were prepared by polymerase change reaction with a forward primer labeled with a biotin molecule, a reverse primer labeled with a digoxigenin molecule, and lambda-phage genome as the template.

The protein constructions were carried out using the protocol similar to the one described by Alexander *et al.* (*19*). The wild-type CaE (UniProtKB: P06495) gene was purchased from Integrated DNA Technologies gene block. The constructs of wild-type and truncated proteins (CaE, CaE^12^, CaE^123^, and CaE^234^) were inserted into a plasmid with flanking Avi and ybbR tags, with a serine-glycine linker after the AviTag. All constructs were verified by plasmid sequencing. Wild-type CaE and truncation proteins were expressed in BL21 (DE3) Competent *Escherichia coli* cells (New England BioLabs, catalog no. C2527). One micromolar recombinant BirA, 25 μM biotin, 5 mM adenosine triphosphate, and 5 mM Mg(OAc)_2_ were used for N terminus biotinylating. Then, the biotinylated proteins were purified using standard elution procedures (Pierce). All purified protein were concentrated with Amicon centrifugal filters (Merck Millipore, 10-kD MWCO) and stored at −80°C.

### AFM characterization

The monomeric and dimeric DNA origami samples were characterized in air with MultiMode 8 atomic force microscope (AFM) with NanoScope V Controller (Bruker Inc.). Each sample of 3 μl was deposited on a freshly cleaved mica surface and was left to adsorb on the surface for 3 min. The mica surface was slowly rinsed with water for three times (each time with 100 μl of water). Then, the mica surface was dried with a mild air stream by an ear-washing bulb and was imaged in tapping mode. All AFM images were analyzed by NanoScope Analysis v1.50.

### Optical tweezers tethers formation

In the optical tweezers geometry, a wild-type or truncated protein was tethered between two DNA-origami or dsDNA handles. C terminus of wild-type and truncated proteins were biotinylated using the enzymatic reaction of coenzyme A (CoA)–biotin (New England BioLabs, catalog no. S9351) and ybbR-tag on the purified protein and phosphopantethein transferase Sfp in 50 mM Hepes (pH 7.5) and 10 mM MgCl_2_ for 1.5 hours at 30°C. The excess CoA-biotin molecules could be removed using Amicon centrifugal filters (Merck Millipore, 3-kD MWCO). The hereinbefore tagging methods provide two biotin attachment points near the C and N terminus of the wild-type and truncated CaE proteins ready for the attachment with the polystyrene beads.

The biotin-/digoxigenin-labeled DNA-origami or dsDNA handles were deposited on bovine serum albumin–passivated anti-digoxigenin–coated 1-μm beads and diluted in 1 × polymix buffer containing 20 mM Hepes-KOH (pH 7.5), 5 mM MgCl_2_, 0.5 mM CaCl_2_, 5 mM NH_4_Cl, 95 mM KCl, 1 mM spermidine, 8 mM putrescine, and 0.1 mM dithiothreitol supplemented with 10 mM NaN_3_, which works as an oxygen scavenger to prevent the formation of reactive singlet oxygen in the optical tweezering experiment. The sample of DNA-origami or dsDNA-coated beads was incubated with streptavidin and then divided into two aliquots. One aliquot was incubated with biotin-labeled proteins at room temperature. These samples were stored on ice until injection into the microchamber of the optical tweezers instrument.

### Optical tweezers measurements

Force spectroscopy measurements were conducted on a dual-trap optical tweezers instrument (*33*) using a solid-state 1064-nm laser, and the trap stiffness was set to 0.25 pN/nm. The experiments were conducted at ambient temperatures in 1 × polymix buffer with azide. For ease of interpretation, single-molecule force spectroscopy experiments were done in the presence of saturating calcium concentration (0.5 mM Ca^2+^). Data collected without calcium showed a shift to an unfolding transition centered at 5 pN and a single, low-force refolding transition that corresponds to the entire chain collapse, instead of domain-wise folding. The results in the absence of Ca^2+^ are consistent with NMR data describing the formation of a molten globule state under these conditions (*15*). The tweezers could trap a bead with protein samples and a bead just with DNA-origami or dsDNA handle. Tethers were formed through bringing the two beads in close proximity to each other and pulling them apart until a connection formed. To ensure that the tether was formed with single molecules, the contour length and curvature of formed tether were calculated and recorded.

In the passive mode (constant trap position) experiments, the traps remain stationary while the protein undergoes folding/unfolding transitions. All equilibrium data were measured in passive mode to avoid artifacts of missed folding transitions and depressed apparent folding rates (*34*).

### Data process and analysis

After a tether was identified, the force was raised to the force of interest. During data analysis, the time between raising the force and tether rupture was recorded. Raw data were collected and analyzed at 2.5 kHz and filtered to 100 to 250 Hz for further analysis and display. Data were collected with a LabVIEW custom interface as previously described (*33*) and analyzed using Matlab and Python.

Hidden Markov model (*35*) based on open-source Python package hmmlearn v0.2.7 was used to analyze the optical tweezers data collected in passive mode. The equilibrium kinetics data and lifetime fitting data were analyzed with Matlab. The estimates of average extension characterizing each state and the states transition probabilities, as well as confidence intervals that characterize the uncertainty in these values were produced. Fits were performed with varying numbers of states, and the best fit was selected on the basis of the lowest Bayesian Information Criterion (*36*). The transition rates were calculated through fitting a cumulative density function to fit lifetimes (*37*).

### Force versus extension curves

The elasticity of the DNA origami transducers was modeled using an extensible freely jointed chain with two elements (Stretch modulus *K* = 1 nN and contour length *L* = 600 nm)

$$F_{\text{eFJC}}\left(x\right)=k_{B}T\;\frac{2\;\left(\frac{x}{L}-\frac{F}{K}\right)}{L\left[1-{\left(\frac{x}{L}-\frac{F}{K}\right)}^{2}\right]}$$ (3)

The value of the stretch modulus *K* was obtained from an independent experiment in which only a DNA-origami transducer was pulled.

To model the elasticity of unfolded polypeptide, a worm-like chain model with persistence length $P_{p}$, and contour length $L_{p}$, and protein extension p was used. A persistence length $P_{p}$ of 0.7 nm for the protein as reported previously.

$$F_{\mathrm{WLC}}\left(p\right)=\frac{k_{B}T}{P_{p}}\left[\frac{p}{L_{p}}+\frac{1}{4{\left(1-\frac{p}{L_{p}}\right)}^{2}}-\frac{1}{4}\right]$$ (4)

### Structural analysis

Proteins undergo a complex folding process facilitated by a dynamic network of noncovalent interactions, including hydrogen bonds, Van der Waal forces, hydrophobic interactions, and ionic interactions. Thus, characterizing these interactions is essential to understand a protein’s folding behavior and functions. In our analysis, we used ProteinTools, a toolkit to identify the hydrophobic clusters, hydrogen bond networks, salt bridges, and contact maps of protein, to analyze the structures data from NMR and AlphaFold.

Hydrophobic clusters are typically secluded within the interior of the protein, away from the surrounding water, enhancing the stability of high-energy partially folded states. Hydrogen bond networks illustrate the electrostatic interactions among residues, crucial for maintaining the correct geometry and orientation of the protein backbone, thus ensuring protein stability and folding. ProteinTools computes hydrogen bond networks among side chains through the examination of nitrogen and oxygen donors and acceptors within a spatial distance of 2.5 Å and having an angle exceeding 120°. Ionic interactions between oppositely charged residues, forming salt bridges, also contribute to protein stability. ProteinTools aids in the identification of salt bridge networks and calculates charge segregation parameters, κ and fraction of charged residues (FCR) (*38*). A salt bridge is the combined results of a hydrogen bond and an ionic bond, representing a substantial force in biological systems and the most commonly observed contribution to the stability of misfolded protein conformations. κ serves as a metric for measuring the degree of charge segregation within a sequence, while FCR represents the proportion of charged residues within the sequence. These values can be used to predict the compactness of proteins. A protein contact map, often depicted as a matrix, represents residue-residue contacts within a distance threshold. Analyzing the patterns of contacts in an inter-residue contact map provides insight into the spatial arrangement of residues in proteins and its correlation with stability and folding.

### AlphaFold analysis

Single-molecule force spectroscopy reveals crucial details about the protein folding behavior but does not provide atomistic details of structural rearrangements during folding. Conversely, traditional structural methods only provide information on proteins in their most stable conformations and not about the intermediates that emerge during folding. To complement the information obtained from the force spectroscopy analysis, we use AlphaFold (*6*, *7*) that enables accurate predictions of protein structures with arbitrary sequences and allows exploration of their conformational landscape using techniques such as clustering of multiple-sequence alignments, as demonstrated by AF-Cluster (*25*).

AlphaFold2 (*6*) structural predictions were generated using ColabFold (*39*). AF-Cluster analysis was performed as described in Wayment-Steele *et al.* (*25*). In brief, multiple-sequence alignments (MSAs) for each protein construct were generated using the MMseqs2-based (*40*) routine implemented in ColabFold. These MSA files were then used as input into AF-Cluster Colab available scripts (https://colab.research.google.com/github/HWaymentSteele/AF_Cluster/blob/main/AFcluster.ipynb). The PDB files generated from this analysis were then used for further inspection in ChimeraX (*41*) and structural analysis. The root mean square deviation for structure models with respect to their corresponding canonical AlphaFold prediction was calculated using MDtraj (*42*). Principal components analysis of the internal coordinates of the ensemble of predicted structures was performed using Scikit-learn (*43*). Protein structures were visualized and superimposed using ChimeraX (*41*).

## Supplementary Materials

### This PDF file includes:

Figs. S1 to S12 Supplementary Text Tables S1 to S10 References
