# Supplementary material for: DNA origami–enhanced force spectroscopy and AlphaFold structural analyses reveal the folding landscape of calcium-binding proteins
Source: Sci Adv. 2025 Apr 30;11(18):eadv1962. doi: 10.1126/sciadv.adv1962 (PMC12042886; doi:10.1126/sciadv.adv1962)
Supplement: Supplementary file 1 — Figs. S1 to S12 Supplementary Text Tables S1 to S10 References [file sciadv.adv1962_sm.pdf]

Science Advances
AAAS

Supplementary Materials for

DNA origami–enhanced force spectroscopy and AlphaFold structural analyses reveal the folding landscape of calcium-binding proteins

Honglu Zhang *et al.*

Corresponding author: Honglu Zhang, z.hl@sjtu.edu.cn; Chunhai Fan, fanchunhai@sjtu.edu.cn; Carlos Bustamante, carlosb@berkeley.edu

Sci. Adv. **11**, eadv1962 (2025)
DOI: 10.1126/sciadv.adv1962

This PDF file includes:

Figs. S1 to S12
Supplementary Text
Tables S1 to S10
References

Supplementary Figures and Text

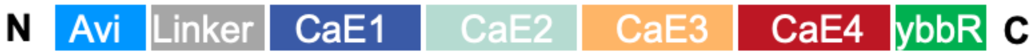

Fig. S1. The sequence contains full-length calerythrin (CaE), flanked by Avi tag at the N-terminus and ybbR tag at the C-terminus. CaE1 (dark blue), CaE2 (light blue), CaE3 (yellow) and CaE4 (red) stand for EF1, EF2, EF3 and EF4 hands.

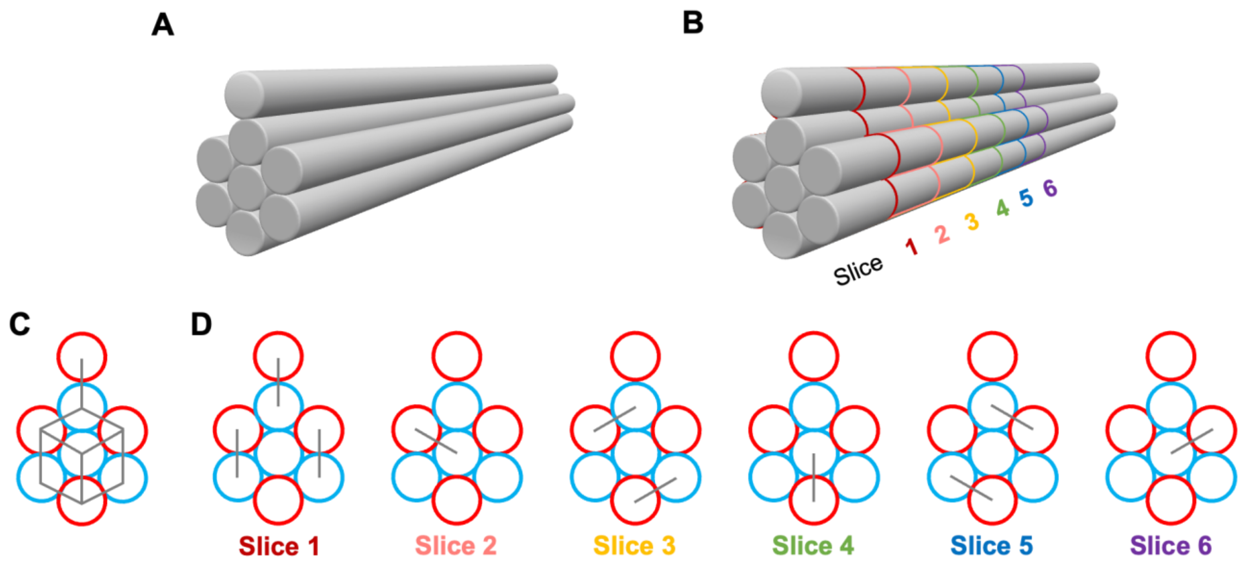

Fig. S2. The geometry of DNA origami of hexagonal lattice, which is the most densely packed pattern. (A) and (C) Schematic illustration and cross-sectional view of single DNA origami assembled from M13mp18 scaffold and 183 staple strands. (B) and (D) Cross-sectional slices for eight-helix hexagonal lattice. The spacing length between neighboring slices is 9 bp. (C) and (D) The bundles are color-coded to indicate the polarity (5'end to 3' end) of the scaffold strand. The scaffold strand threads through DNA double helices coded in blue color and their counterparts coded in red with opposite polarities. Staple strand-crossover (gray color) patterns of each cross-sectional slice.

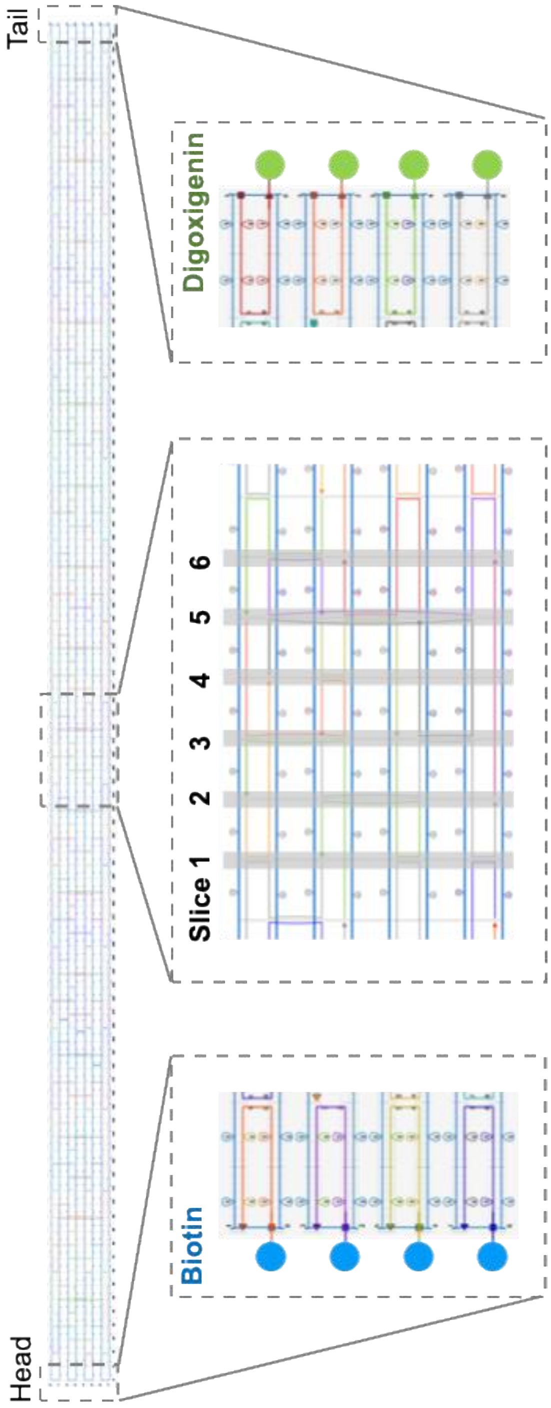

Fig. S3. The sequence of a single DNA origami design using Cadnano2. The long blue strand represents the scaffold M13mp18 DNA. The short, colored strands are staples with arrows indicating 5’ to 3’ direction. Insets, Biotin-labeled (blue solid circles) and digoxigenin-labeled (green solid circles) staple strands are positioned at the head and tail of the DNA origami structure. The middle inset shows a zoomed-in figure of a representative slice pattern, as shown in Fig. S2.

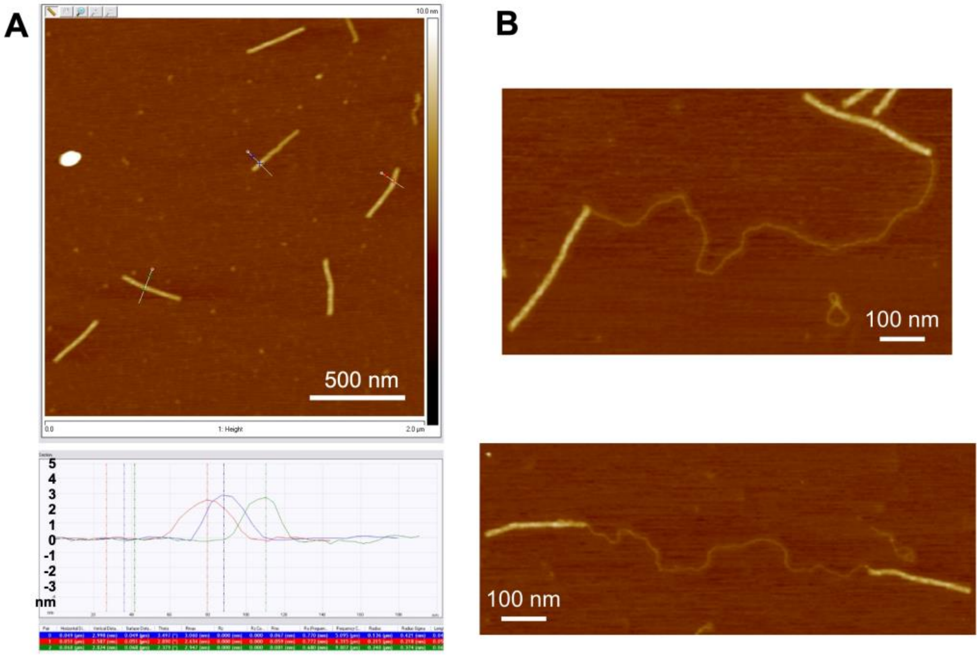

Fig. S4. AFM characterization of 8-helix DNA origami. (A)The AFM images (upper panel) and height measurement (lower panel) of single DNA origami. The sample was imaged with AFM tapping mode in air environment. The heights of DNA origami structures were measured at 2.998 nm (blue line), 2.587 nm (red line) and 2.824 nm (green line). (B) The AFM images of the conjugations of DNA origami with a dsDNA strand. A 3kbp-long dsDNA is flanked by two DNA origami structures.

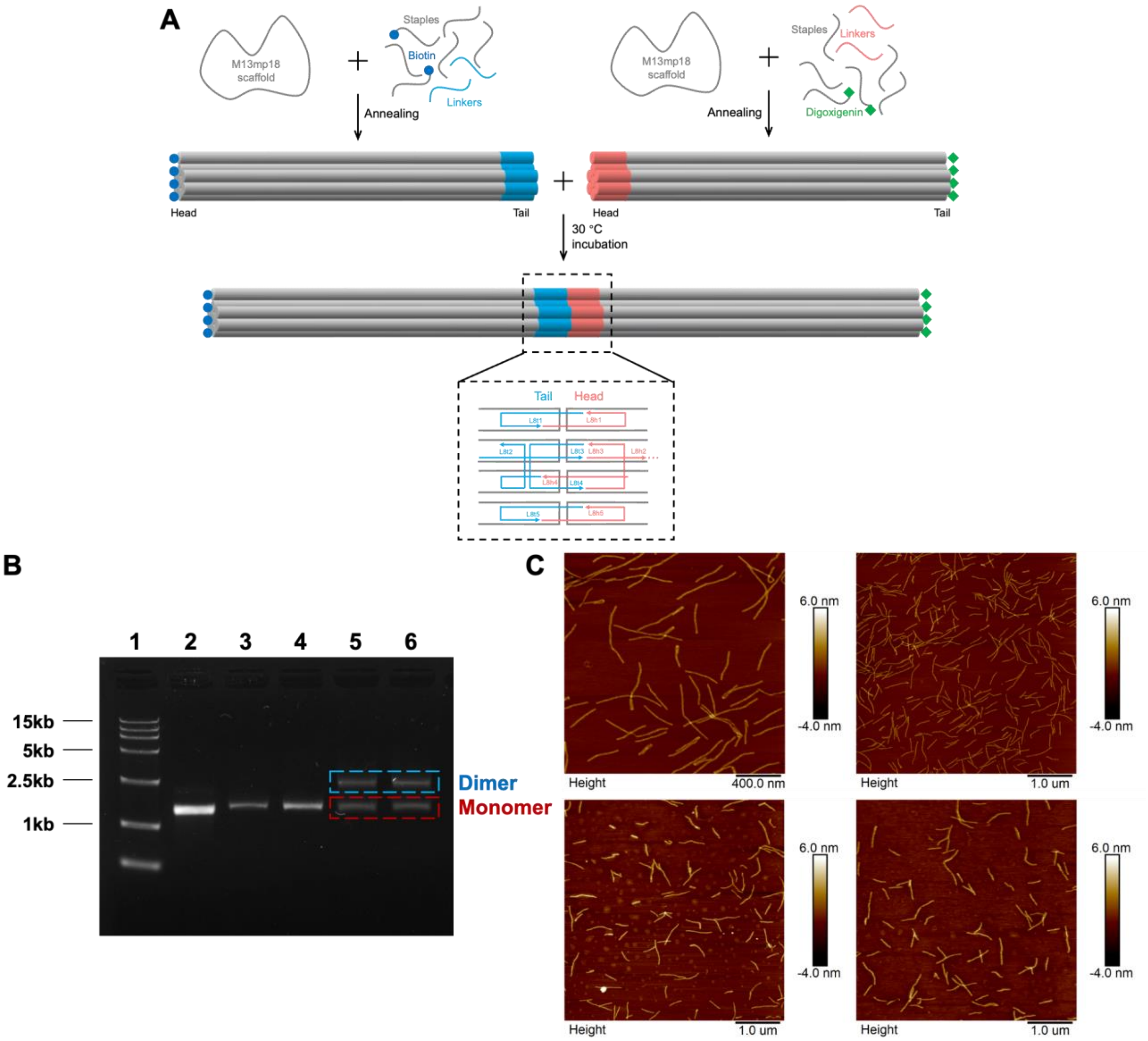

Fig. S5. Scheme and experimental characterization of the hierarchical assembly of origami monomers. (A) Scheme of the hierarchical assembly of origami monomers. Scaffold M13mp18 strand was mixed with staple strands and linker stands (blue or red color) in 1x TAE-Mg^{2+} buffer with a ratio of 1:10 from 95 °C to room temperature in a rate of 1 °C min^{-1}. The sequences of strands are listed on Table S4. One origami monomer was assembled with biotin-labeled staple strands at head section and blue linkers capped tail section. The other monomer was assembled with red linkers capped head section and digoxigenin-labeled staples at tail section. These two origami monomers were purified by agarose gel electrophoresis, respectively and then mixed together to incubate at constant temperatures of 37 °C. The blue and red linkers hybridized with each other and no unhybridized single-stranded spacer sequence between two origami monomers. (B) Agarose gel electrophoresis of DNA origami dimers in 1× TAE/Mg^{2+} buffer. Lane 1, DL 15,000 DNA maker; lane 2, M13mp18 ssDNA; lane 3, 8-helix DNA origami monomer A (with tail linker strands); lane 4, 8-helix DNA origami monomer B (with head linker strands); lane 5, 8-helix DNA origami dimer after connection reaction at 37 °C for 5 min (yield 68%); lane 6, 8-helix DNA origami dimer after connection reaction at 37 °C for 10 min (yield 72.8%). (C) AFM characterization of 8-helix DNA origami dimers after connection reaction at 37 °C for 10 min (yield 76.9%).

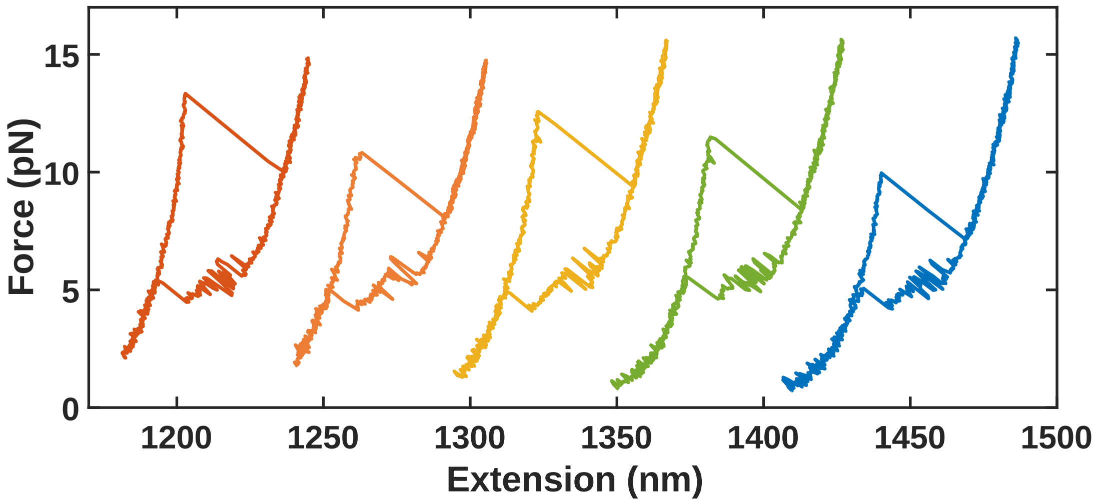

Fig. S6. Representative force-extension curves (stretching and relaxing cycles) of CaE with DNA origami handles.

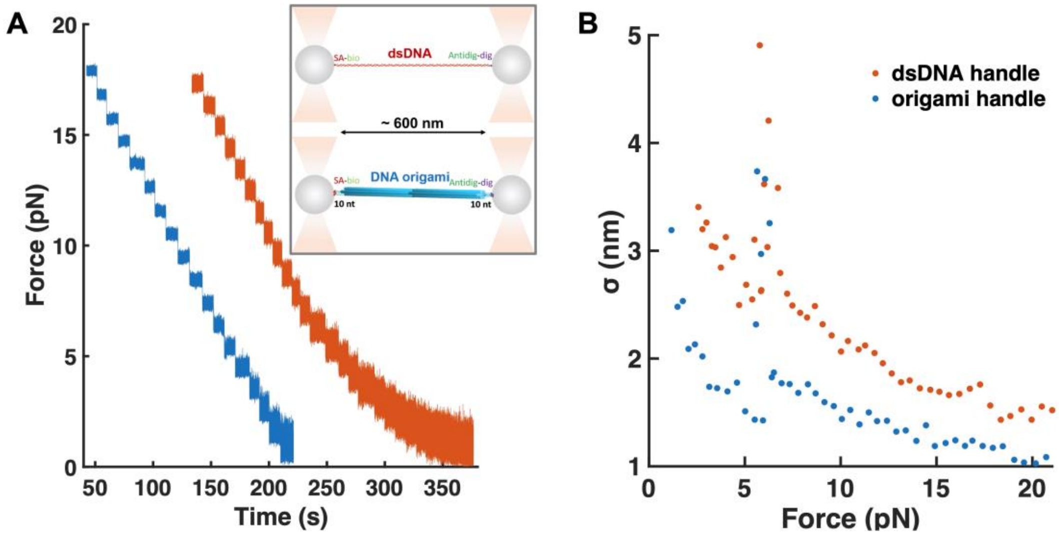

Fig. S7. Comparison of force-time traces and noise amplitudes in tweezing CaE with DNA origami vs. dsDNA handles. (A)Force-time traces with DNA origami (blue) or dsDNA (red) handles. (B) Force-dependent noise amplitudes (root mean square displacement of trapped beads, σ) of tweezing CaE with DNA origami (blue) or dsDNA (red) handles.

Supplementary Text: Increase in stiffness of DNA origami transducer reduces noise readouts

Based on the force extension curves, we estimate the stiffness of tethering geometry at ~5 pN with the DNA origami transducers to be ~1.3 pN/nm compared to ~0.15 pN/nm for conventional dsDNA. This increase in stiffness is in accordance with theoretical predictions from rigid tube theory, where the increased number of structural beams (N=8 helices for our handle design) contributes to the stiffness. Given these values we can compute the predicted noise using the following equation:

$$\Delta x_{RMS} = \frac{\Delta F_{RMS}}{\kappa_{trap}+\kappa_{molecular}} = \frac{2\sqrt{k_B T \gamma B}}{\kappa_{trap}+\kappa_{molecular}} \qquad \text{(S1)}$$

where ΔF_{RMS} is the noise in the force readout, κ_{trap} is the trap stiffness, $\kappa_{molecular}$ is the molecular tether stiffness, B is the bandwidth of the measurement, and γ is the drag coefficient of the bead.

Given the experimental trap stiffness (κ_{trap}=0.2 pN/nm) and the estimated molecular stiffness ($\kappa_{molecular}$) values, expression (1) predicts a nearly four-fold decrease in noise relative to that observed when replacing the DNA handles with the DNA origami constructs. However, our experimental data show only a two-fold decrease in noise. We speculate that this discrepancy may be due to the added flexibility of the ssDNA linker connecting biotin/digoxigenin and DNA origami, and of the linker strands bridging the two DNA origami monomers in the dimer. These factors introduce some additional compliance into the system, reducing the expected noise suppression.

In addition to noise reduction, we also observe a significant increase in signal. The increased stiffness of the DNA origami transducers allows a greater proportion of the tether's extension to be measured. For example, at ~5 pN, only 43% of the signal is effectively measured using conventional dsDNA handles, while 86% of the signal is captured with DNA origami transducers. This signal amplification aligns with theoretical expectations, as shown by the following expression:

$$\Delta x_{signal} = \frac{\kappa_{molecular}}{\kappa_{trap}+\kappa_{molecular}} \Delta x \qquad \text{(S2)}$$

However, as the protein unfolds, its reduced stiffness ($\kappa_{polypeptide}$) becomes more dominant, and the effective molecular stiffness ($\kappa_{molecular}$) is given by:

$$\kappa_{molecular} = \frac{1}{\frac{1}{\kappa_{handles}}+\frac{1}{\kappa_{polypeptide}}} \qquad \text{(S3)}$$

Thus, the signal-to-noise ratio and the noise suppression by DNA origami transducers are both reduced once protein unfolding has occurred (Fig. S7B).

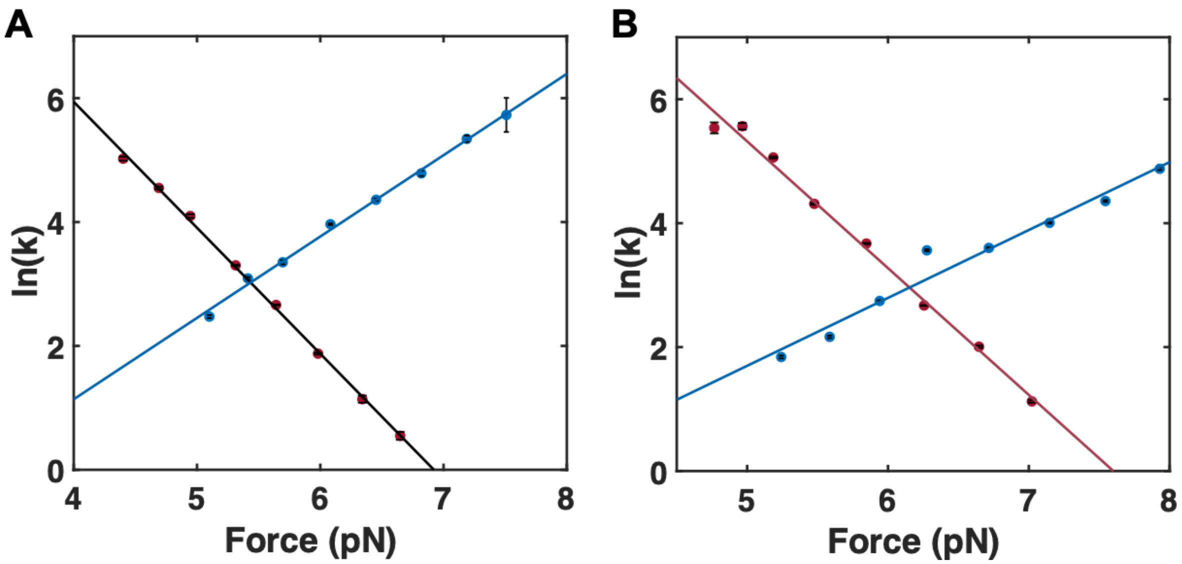

Fig. S8. Representative force-dependent unfolding and refolding transition kinetics of CaE (A) and CaE^{234} (B). The effect of force on the transition rates between U and I2 (A), as well as between U and I (B), is plotted. For CaE (A), the folding and unfolding rates between U and I2 decrease and increase, respectively, with the magnitude of the force applied to the protein. The intersection of the distributions for folding and unfolding rates occurs at approximately 5.5 pN, which represents the force at which I2 spends 50% of its time folded and 50% unfolded.

Supplementary Text: *Cis-Trans* Pro103 Isomerization (Fig. S9)

Understanding this mechanism can be achieved through a detailed structural analysis. Both NMR and AlphaFold structural diagrams of CaE reveal an interesting feature: there is a bend near the linker region (residues #91-99), which connects the N and C domains of the protein (Fig. S9). This bend coincides with the presence of a proline residue (Pro) at position #103. Previously reported research has shown that alpha helices that contain prolines tend to adopt kinks 30- to 100-fold more frequently than helices not containing proline, due to the tendency of this residue to adopt the *cis* isomer form (*44*). This is particularly notable when the amino acid preceding proline is glycine (Gly) (*45*). In the case of CaE, a Gly is found at position #102. The increased tendency of Pro103 to adopt the *cis* conformation in the linker region, may impede the direct folding of EF2 or EF12 onto the pre-folded C domain to arrive at the native protein structure. As a result, this proline isomerization can lead to the formation of two distinct platforms composed of EF234, one with the proline in *cis* conformation (intermediate I1') that does not allow for the folding docking of EF1 and another with the proline in the correct *trans* configuration (intermediate I1) that permits the attainment of the fully folded CaE.

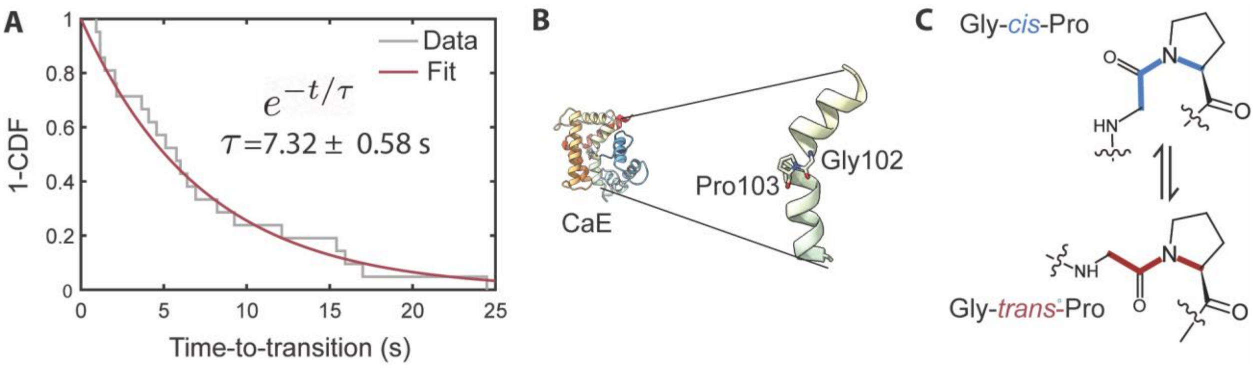

Fig. S9. *Cis-Trans* Pro103 Isomerization. (A) Complementary Cumulative Distribution Function (1-CDF) of the time measured to attain the folded state for the traces where the transition to the folded state was observed as in Fig. 4A. The 1-CDF fits well to a single exponential, suggesting a unique molecular event responsible for stabilizing the transition to the folded state. (B) The linker bend induced by the Pro103 and Gly102 isomerization. (C) Conformational transition between *cis* and *trans* states**.**

The requirement of a pre-folded C domain for the attainment of the native state observed here has been found in other CaM-like EF-hand proteins, such as parvalbumins, a three-EF hand protein subfamily that is thought to originate from an ancestral CaM-like tandem domain protein that lost the first EF-hand of the N domain (*46*, *47*). The second, unpaired EF hand of parvalbumin folds onto the pre-folded calcium bound C domain, helping to stabilize it. In the case of CaE, the non-functional EF2 is found to behave like a peptide that interacts transiently with the folded C domain possibly depending on the isomerization of Pro103. This transient behavior continues until the on-pathway intermediate I1 (EF234 with Pro103 in the *trans* configuration) forms, which in turn leads to the quick association of the calcium-bound EF1 and the attainment of the stable native form of CaE.

Supplementary Text: Conformational analysis using AF-Cluster (Fig. S10)

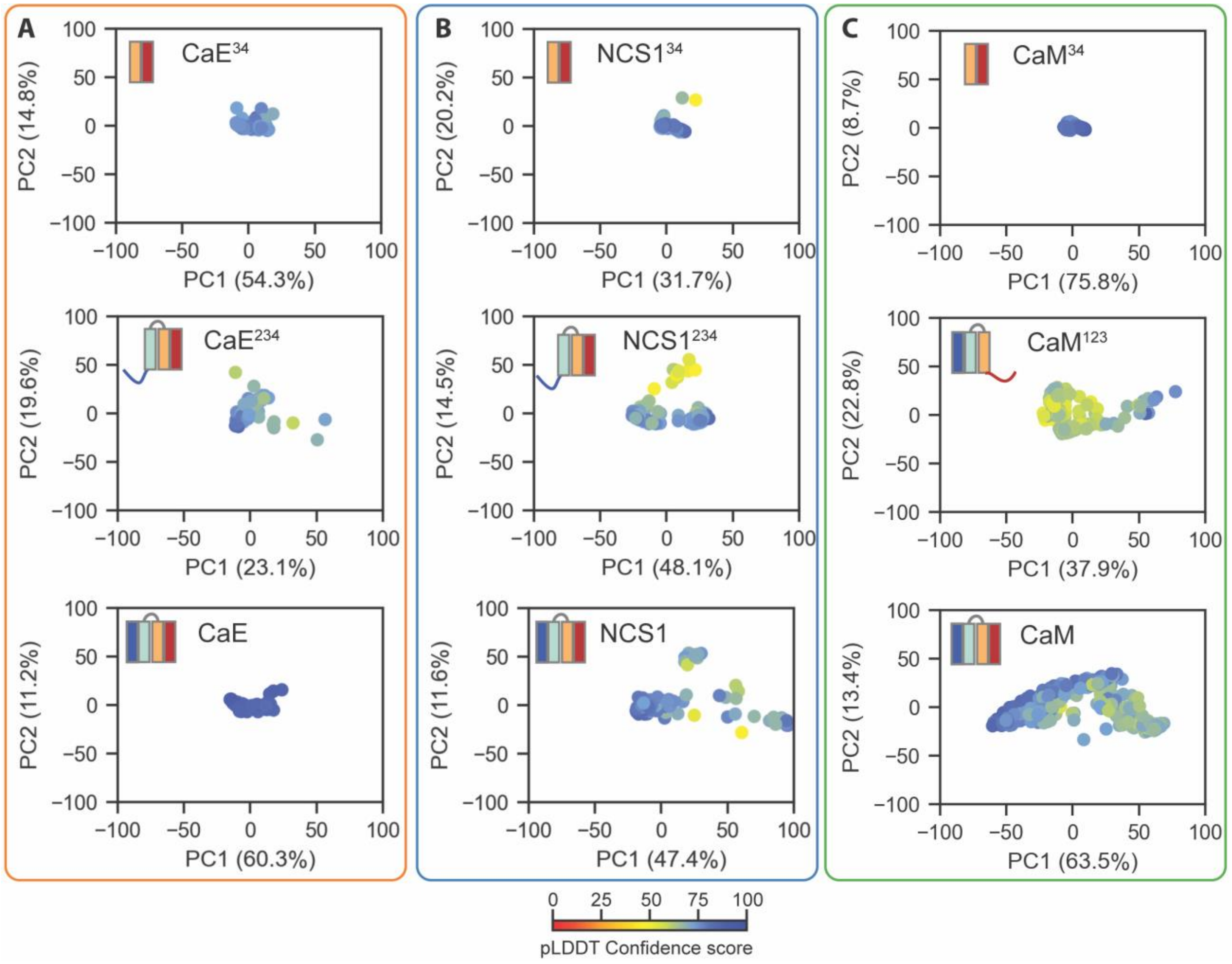

Fig. S10. Principal Component Analysis (PCA) of the internal coordinates of the ensemble of states predicted using AF-Cluster. A score plot of the first two principal components (PCs) of CaE (A), NCS1 (B), CaM (C), and their truncated proteins is shown. The spread displayed in these plots indicates conformational heterogeneity.

Supplementary Text: Hydrophobic interactions, hydrogen bond and salt bridge networks analysis of calcium-binding proteins (Fig. S11-S12)

The structural analysis data are presented in Fig. S11 (NMR) and Fig. S12 (AlphaFold2). For CaE, the analysis of hydrophobic interactions reveals a small cluster of only two residues and a large cluster comprising 24 residues in NMR and 26 residues in AlphaFold2, comprising a total area of 2892.49 Å2 (2755.08 Å2, AlphaFold2). The large cluster mainly spans residues of the EF2 hand, the first helix region of EF3 hand, and the second helix region of EF4 hand. The area per residue is 44.5 Å2, and there are 65 total contacts among the residues. We compared CaE's large hydrophobic clusters with those present in members of the other four-EF-hand families. CaM dis-plays a 5-residue cluster in NMR and 6 in AlphaFold2, whereas NCS1 shows a 7-residue cluster in NMR and 6 in AlphaFold2. All contact residue numbers for these two proteins are less than 10 (Table S5). In contrast, the cluster number in CaE is 24 (26, AlphaFold2). The largest area of hydrophobic clusters in CaM and NCS1 are 674.64 Å2 in NMR and 658.9 Å2 in AlphaFold2 and 756.87 Å2 in NMR and 746.45 Å2 in AlphaFold2, respectively, significantly smaller than the hydrophobic clusters in CaE (2892.49 Å2 in NMR and 2755.08 Å2 by AlphaFold2). The higher order of contacts observed in CaE are likely responsible for the additional mechanical stability of this protein displayed in the larger rupture forces and the hysteretic behavior between the un-folding and refolding force spectroscopy trajectories.

In the NMR data, the proteins CaM, NCS1 and CaE exhibit five, four and three hydrogen bond networks respectively, and these networks show minimal differences among the three proteins. However, when analyzing their AlphaFold2 structures, differences are observed in their hydrogen bond networks. Specifically, CaE and NCS1 exhibit 14 and 19 hydrogen bond networks, respectively, compared to the 11 networks observed in CaM. Similarly, in terms of salt bridge networks, CaM, NCS1 and CaE contain one, three and two networks, respectively, in the NMR data. However, their salt bridge networks exhibit notable variations when compared in the AlphaFold2 data. Both CaE and NCS1 proteins display seven salt bridge networks, whereas CaM only possesses two networks.

Further analysis reveals that within CaE, nine out of the total 14 hydrogen bond networks, as well as four of the total seven salt bridge networks, are located within the corresponding EF hands, serving to stabilize the local structural integrity of EF hand. In the case of NCS1, 14 of the total 19 hydrogen bond networks, and five out of the total seven salt bridge networks, span EF3 and EF4 hands. Notably, the largest salt bridge network, labeled as #6, consists of three residues and extends across the internal EF4 hand. These structural interactions contribute to the formation of intermediate EF3 and EF34 during the folding transitions of NCS1. In contrast, the AlphaFold2 data of CaM reveals that all 11 hydrogen bond networks and two salt bridge networks span either the EF1 hand and EF2 hand or the EF3 hand and EF4 hand. These interactions occur within the individual domain and do not involve any interactions between the N- and C-domains. These interaction networks explain the presence of intermediate state EF12 and EF34 during the folding transitions of CaM.

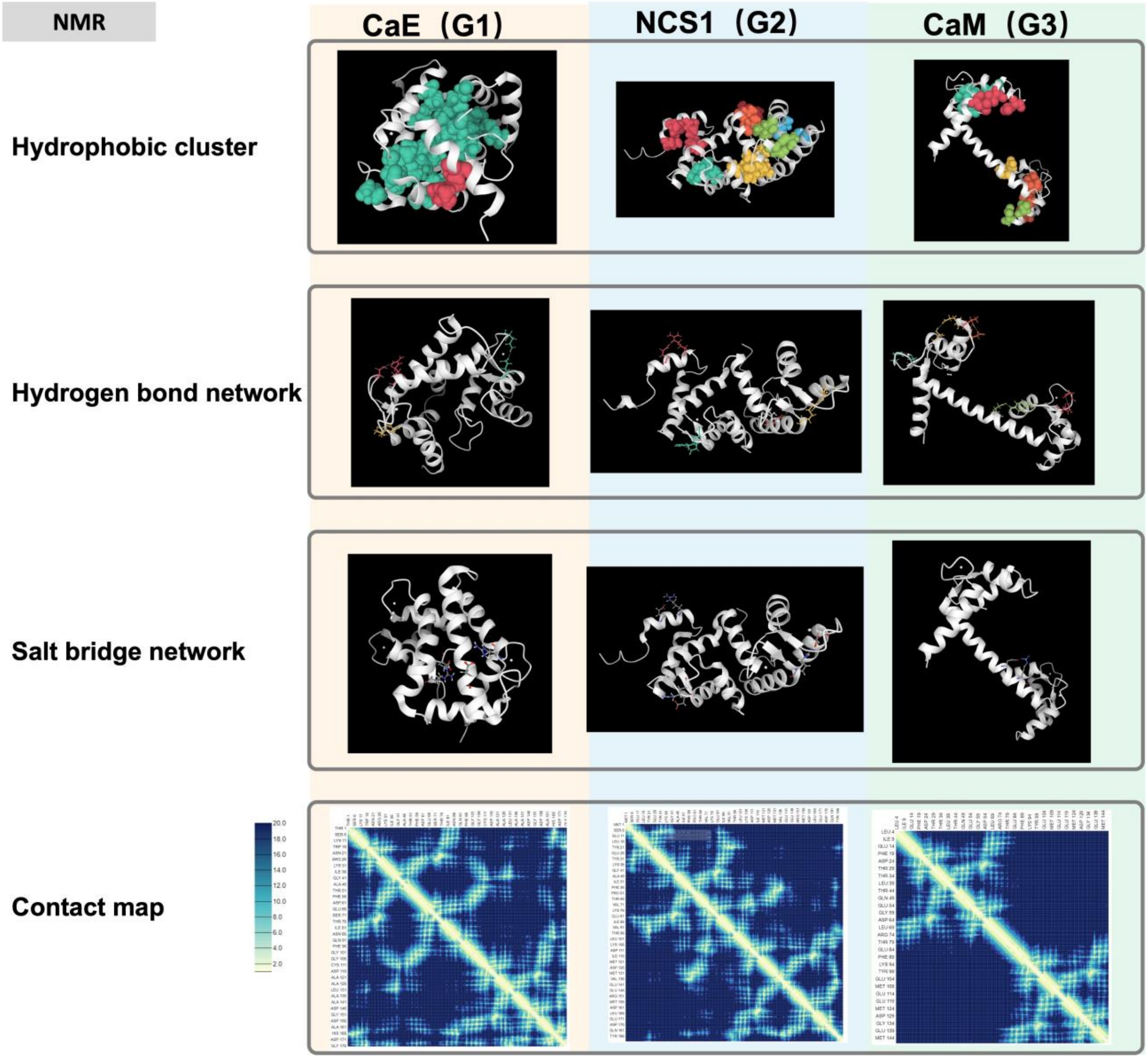

Fig. S11. Hydrophobic cluster, hydrogen bond network, salt bridge network and contact map analysis of proteins CaE, NCS1 and CaM (NMR data).

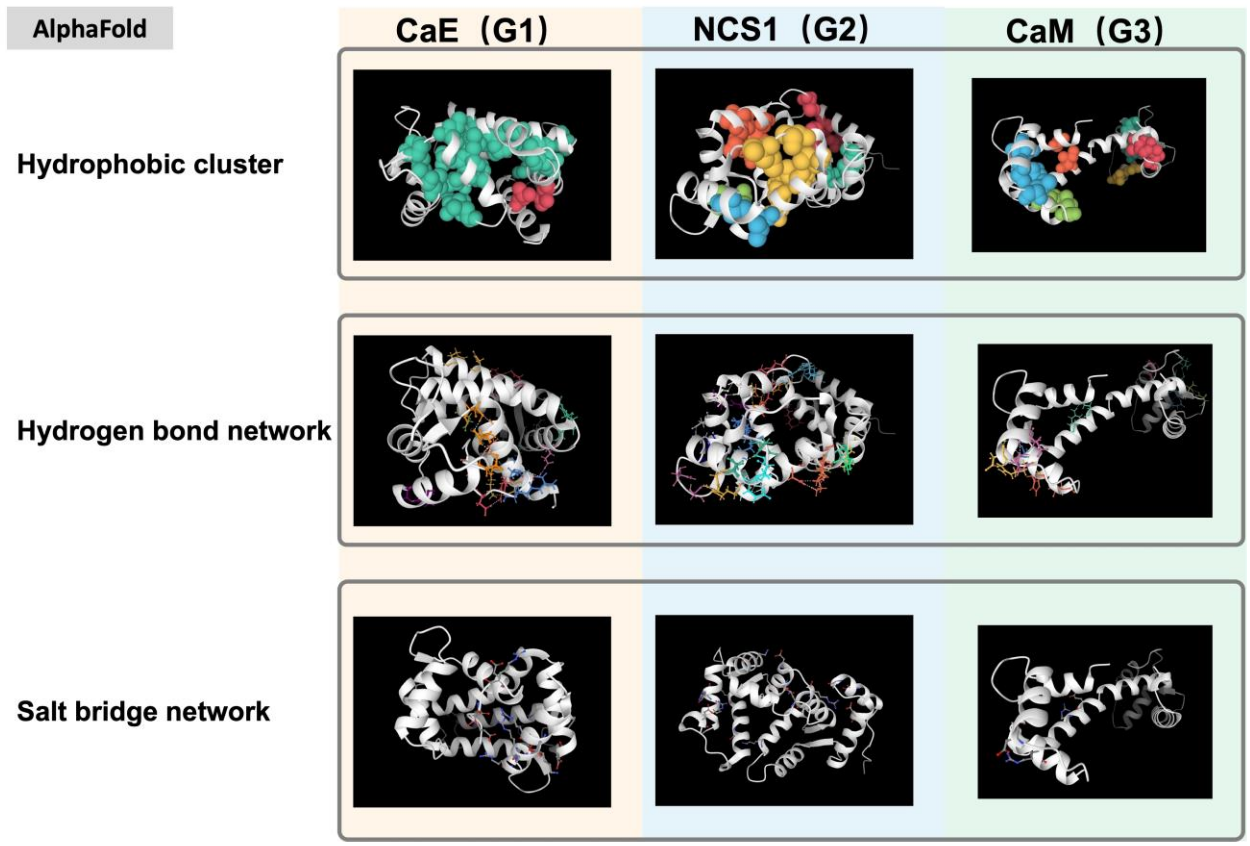

Fig. S12. Hydrophobic cluster, hydrogen bond network and salt bridge network analysis of proteins CaE, NCS1 and CaM (AlphaFold data).

EF hand	ID positions
**1**	5-40
**2**	45-90
**3**	100-134
**4**	134-169

Table S1. Sequence positions of EF hands within the CaE protein.

Structures	ID positions
**Helix**	5-16
**Beta strand**	23-25
**Helix**	28-40
**Beta strand**	45-47
**Helix**	48-68
**Helix**	78-89
**Beta strand**	90-92
**Helix**	94-111
**Beta strand**	118-121
**Helix**	122-131
**Helix**	136-146
**Beta strand**	151-155
**Helix**	156-163
**Beta strand**	167-169

Table S2. Sequence positions of helices and beta strands within the CaE protein.

5'	3'	Sequence
0[23]	1[39]	GGCAAAGAAGCTAAATCGGTTGTACCAAAAACATTA
0[47]	2[32]	TTAACATCCAATAAATCACTTCCTGTATTAAAATTC
0[71]	1[87]	GCATCAATTGCCTTTATTTCAACGCAAGGATAAAAA
0[95]	2[80]	TCATTTGGGGCGCGAGCTGATTCTCCGAAAAACAGG
0[119]	1[135]	TGGTCAATAGCAATGCCTGAGTAATGTGTAGGTAAA
0[143]	2[128]	GTTTGACCATTAGATACATGTAGATGGCGTAAAACT
0[167]	1[183]	TCCCAATTCACAGTCAAATCACCATCAATATGATAT
0[191]	2[176]	GAAGTTTCATTCCATATATATCGGCCTATCAGGTCA
0[215]	1[231]	AAATATGCACCGGAGAGGTGAATTTCTTAAACAGCT
0[239]	2[224]	ATATAATGCTGTAGCTCAAGAAAATACCGGTTTATC
0[263]	1[279]	GCGGATGGCCAACAACCATCGCCCACGCATAACCGA
0[287]	2[272]	TGCTCCTTTTGATAAGAGGAATAAGTTTTTCACGTT
0[311]	1[327]	AGGTCAGGAAGTTAAAGGCCGCTTTTGCGGGATCGT
0[335]	2[320]	CGAACCAGACCGGAAGCAAAAGACAAATTCAGCGGA
0[359]	1[375]	TATCGCGTTGAACGAGGGTAGCAACGGCTACAGAGG
0[383]	2[368]	TTAAGAGGAAGCCCGAAAGAAATTATTGACGTTAGT
0[407]	1[423]	GAAGCAAAGGGAAGTTTCCATTAAACGGGTAAAATA
0[431]	2[416]	AGGTCTTTACCCTGACTAATTAGAGCCCCACAGACA
0[455]	1[471]	AACGAGAATTAAAACGAAAGAGGCAAAAGAATACAC
0[479]	2[464]	CCCTCAAATGCTTTAAACGTCACCAATTGTACCGTA
0[503]	1[519]	GAATCGTCAATTATACCAAGCGCGAAACAAAGTACA
0[527]	2[512]	TTAGACTGGATAGCGTCCTTTGCCTTTACCCTCAGA
0[551]	1[567]	TTGCCAGAGATTGTGTCGAAATCCGCGACCTGCTCC
0[575]	2[560]	TAGCGAGAGGCTTTTGCACCCCTTATTGTACTCAGG
0[599]	1[615]	TTTACCAGAACGGTCAATCATAAGGGAACCGAACTG
0[623]	2[608]	GTAAGAGCAACACTATCAACCACCGGAATAAGTGCC
0[647]	1[663]	TAACGCCAAGGTGTACAGACCAGGCGCATAGGCTGG
0[671]	2[656]	ATACCACATTCAACTAATGCCACCACCAGACTCCTC
0[695]	1[711]	GAAAGATTCAAGAACCGGATATTCATTACCCAAATC
0[719]	2[704]	ACTAACGGAACAACATTAGACAGGAGGCCCCTGCCT
0[743]	1[759]	GAAGAAAAAATAAGGCTTGCCCTGACGAGAAACACC
0[767]	2[752]	GGCTCATTATACCAGTCAATCCTCATTAGTTTTAAC
0[791]	1[791]	GAATTACCTTGGTTTAAT
1[40]	5[47]	TGACCCTGTAAAATCCTGTTTGATGGTATAGCTGTTTCCTGTGTG
1[88]	5[95]	TTTTTAGAACTGGCCCTGAGAGAGTTGAGCCGGAAGCATAAAGTG
1[136]	5[143]	GATTCAAAAGGTGGTTTTTCTTTTCACATTAATTGCGTTGCGCTC
1[184]	5[191]	TCAACCGTTAATGGAAGAATCGGCCAAGCTGCATTAATACAGTAC
1[232]	5[239]	TGATACCGAATCAAGAAAACAAAATTAGTAAATCGTCGCTATTAA
1[280]	5[287]	TATATTCGGAGAGGCGAATTATTCATTAGCTTAGATTAAGACGCT
1[328]	5[335]	CACCCTCAGGAAACAATAACGGATTCGTCTGAGAGACTACCTTTT
1[376]	5[383]	CTTTGAGGATTTTCAGGTTTAACGTCATAAATGCTGATGCAAATC
1[424]	5[431]	CGTAATGCCCCTACCATATCAAAATTAAATATATTTTAGTTAATT
1[472]	5[479]	TAAAACACTTCAATATAATCCTGATTGGACCGTGTGATAAATAAG
1[520]	5[527]	ACGGAGATTCCAGAAGGAGCGGAATTATAGAAAAAGCCTGTTTAG
1[568]	5[575]	ATGTTACTTTTATTAATTTTAAAAGTTGCCAACGCTCAACAGTAG
1[616]	5[623]	ACCAACTTTGTATTAGACTTTACAAACCATGTAATTTAGGCAGAG
1[664]	5[671]	CTGACCTTCCTAACAACTAATAGATTACGACAAAAGGTAAAGTAA
1[712]	5[719]	AACGTAACAAAATCAACAGTTGAAAGGCTAATGCAGAACGCGCCT
1[760]	5[767]	AGAACGAGTTCACCTTGCTGAACCTCATAATATCCCATCCTAATT
2[15]	0[8]	ATCAGCTCAGAGCATAAATTAGCAAAA
2[31]	7[23]	GCATTAAATATCATGGTCGGTTCCGAAATCGGCAAATTGAGTGTT
2[47]	4[32]	AATATTTTGGCCAGCTTTTGTAAAACGACGGCCAGT
2[63]	0[48]	ATTTAAATTGCGGGAGAACTACTAATAGTAGTAGCA
2[79]	7[71]	AAGATTGTACAACATACGCAGCAAGCGGTCCACGCTAACGTCAAA
2[95]	4[80]	AAAAGCCCCTGGGAACAATGTGCTGCAAGGCGATTA

2[111]	0[96]	ACCCCGGTTATTTTAAATACCTGTTTAGCTATATTT
2[127]	7[119]	AGCATGTCACTAACTCACCAGTGAGACGGGCAACAGGGAACCCTA
2[143]	4[128]	AACGGTAATGCGCATCGTGAAGGGCGATCGGTGCGG
2[159]	0[144]	CAAACAAGAAGGCCGGAGTGCGAACGAGTAGATTTA
2[175]	7[167]	TTGCCTGAGGTCGTGCCACGCGCGGGGAGAGGCGGTTGGCGAGAA
2[191]	4[176]	ACAAAGGCTCAGGAAGATGGTGCCGGAAACCAGGCA
2[207]	0[192]	GCTATTTTTAAATTAATGACTAAAGTACGGTGTCTG
2[223]	7[215]	AGCTTGCTTCTTGCTTCTATTACATTTAACAATTTCGTGTAGCGG
2[239]	4[224]	TAATTGTATATACATAAAATACCCAAAAGAACTGGC
2[255]	0[240]	GGCTCCAAACGACAATGATTAGAGCTTAATTGCTGA
2[271]	7[263]	GAAAATCTCCATAGCGATTCAATTACCTGAGCAAAATACAGGGCG
2[287]	4[272]	TAATAATTTTATTTTGTCAGATAGCCGAACAAAGTT
2[303]	0[288]	AACTAAAGGCTTGCAGGGTTAGAGAGTACCTTTAAT
2[319]	7[311]	GTGAGAATAAATCATAGGCCTGATTGCTTTGAATACTTAGAATCA
2[335]	4[320]	TTCAACAGTAGGGCGACAAACAATGAAATAGCAATA
2[351]	0[336]	GGATTTTGCACAGCATCGTTAATTCGAGCTTCAAAG
2[367]	7[359]	AAATGAATTAACTATATGGATGAATATACAGTAACAAACGGTACG
2[383]	4[368]	GTCTTTCCACATTAAAGGATATCAGAGAGATAACCC
2[399]	0[384]	ACGATCTAATTTTCATGACGGATTGCATCAAAAAGA
2[415]	7[407]	GCCCTCATAACTTTTTCATTTGCACGTAAAACAGAAAAAAGAGTC
2[431]	4[416]	TGTAGCATTAGCAAAATCGACGGGAGAATTAACTGA
2[447]	0[432]	AGTACAAACGCACCAACCGACCATAAATCAAAAATC
2[463]	7[455]	ACACTGAGTTGAAATACCTTTGGATTATACTTCTGAGTAATAACA
2[479]	4[464]	AGGAACCCAGAAACCATCATGAAAATAGCAGCCTTT
2[495]	0[480]	AGGGATAGCCCCCCAGCGTAAATATTCATTGAATCC
2[511]	7[503]	GCCACCACCCATAATTACTCATCATATTCCTGATTAATATCCAGA
2[527]	4[512]	AGAACCGCCAGCGTCAGACCATATTATTTATCCCAA
2[543]	0[528]	CTCAGAACCGCCTGATAAGGGGTAATAGTAAAATGT
2[559]	7[551]	AGGTTTAGTCAGTATAAATGAGTAACATTATCATTTATACCTACA
2[575]	4[560]	TGTATCACCAGCGTTTGCCAACGCTAACGAGCGTCT
2[591]	0[576]	AGTATAGCCGAGGCGCAGCGACGATAAAAACCAAAA
2[607]	7[599]	GTCGAGAGGCAACGCCAAAATTCGACAACTCGTATTTCACCAGTC
2[623]	4[608]	ACCAGGCGGACCGCCTCCAATCAAGATTAGTTGCTA
2[639]	0[624]	AGCGGGGTTCAGATGAACAAGGAATTACGAGGCATA
2[655]	7[647]	AAGAGAAGGATAAAGTACGAGCCGTCAATAGATAATCCCTTCTGA
2[671]	4[656]	AAGAGGCTGCTCAGAGCCGAACGCGAGGCGTTTTAG
2[687]	0[672]	CTGAAACATAATCTTGACATCAGTTGAGATTTAGGA
2[703]	7[695]	ATTTCGGAACATGTTCAGAATTGAGGAAGGTTATCTCTATTAGTC
2[719]	4[704]	AGTTAATGCTTGAGGCAGTACCGCGCCCAATAGCAA
2[735]	0[720]	AACAGTGCCATTCAGTGATCTACGTTAATAAAACGA
2[751]	7[743]	GGGGTCAGTCAAGAAAAAAATATCAAACCCTCAATCCCGAACGAA
2[767]	4[752]	CTGGTAATAAAAGCCAGATACCGCACTCATCGAGAA
2[783]	0[768]	TGATGATACGGCTTGAGATATGCGATTTTAAGAACT
3[8]	0[24]	CGCCATCAAAAATAATTCGCGTCTGGCTACAGGCAA
3[56]	0[72]	TAAATGTGAGCGAGTAACAACCCGTCGGAAAAGGTG
3[104]	0[120]	TGACCGTAATGGGATAGGTCACGTTGGTTTCGCAAA
3[152]	0[168]	TCTGCCAGTTTGAGGGGACGACGACAGACAGTTGAT
3[200]	0[216]	GCCAGCTCGCAGTATGTTAGCAAACGTACATGTTTT
3[248]	0[264]	ATATAAAAGAAACGCAAAGACACCACGGTCATTTTT
3[296]	0[312]	AGAAAATTCATATGGTTTACCAGCGCCAACTCCAAC
3[344]	0[360]	TTGAGGGAGGGAAGGTAAATATTGACGGACTTCAAA
3[392]	0[408]	ACCGTCACCGACTTGAGCCATTTGGGATTATAGTCA
3[440]	0[456]	ACCATTACCATTAGCAAGGCCGGAAACAGTTCAGAA
3[488]	0[504]	ACCGTAATCAGTAGCGACAGAATCAAGAATACTGCG
3[536]	0[552]	GTTTTCATCGGCATTTTCGGTCATAGCAAAGAAGTT
3[584]	0[600]	ATAATCAAAATCACCGGAACCAGAGCCTAACCCTCG
3[632]	0[648]	GCCACCCTCAGAACCGCCACCCTCAGAGCAGATACA

3[680]	0[696]	ACCACCACCAGAGCCGCCGCCAGCATTTTACAGGTA
3[728]	0[744]	TTGGCCTTGATATTCACAAACAAATAAGGACGTTGG
3[776]	3[791]	GCAGTCTCTGAATTTACC
4[31]	2[16]	GCCAAGCTTGCATGCCTGGAATTCGTATTTTGTTAA
4[79]	2[64]	AGTTGGGTAACGCCAGGGAATTCCACATAAGCAAAT
4[127]	2[112]	GCCTCTTCGCTATTACGCATGAGTGAGATCATATGT
4[175]	2[160]	AAGCGCCATTCGCCATTCGGGAAACCTAGTCTGGAG
4[223]	2[208]	ATGATTAAGACTCCTTATGTGAATAACTCGAGGGTA
4[271]	2[256]	ACCAGAAGGAAACCGAGGCCTTGAAAACAAAAAAAA
4[319]	2[304]	GCTATCTTACCGAAGCCCATTTATCAAGAAAGGAAC
4[367]	2[352]	ACAAGAATTGAGTTAAGCGGGTTATATTTCTGTATG
4[415]	2[400]	ACACCCTGAACAAAGTCACGCGAGAAAGTTAGCGTA
4[463]	2[448]	ACAGAGAGAATAACATAATTAATGGTTTTCGTCACC
4[511]	2[496]	TCCAAATAAGAAACGATTCACCGGAATCTCATTTTC
4[559]	2[544]	TTCCAGAGCCTAATTTGCAATTCTTACACCGCCACC
4[607]	2[592]	TTTTGCACCCAGCTACAACATATTTAAGTTGATATA
4[655]	2[640]	CGAACCTCCCGACTTGCGTAAGAGAATATTAGGATT
4[703]	2[688]	GCAAATCAGATATAGAAGAATAAACAACCTATTATT
4[751]	2[736]	CAAGCAAGCCGTTTTTATAGTCCTGAAGCCTTGAGT
4[791]	2[784]	CATTCCAAGAATCAATAACATGGCTTT
5[8]	4[8]	ACCGAGCTCCAGGTCGAC
5[48]	3[55]	AAATTGTTATCCGCTCACTTTTCCCAGTCACGACGTCATCAACAT
5[96]	3[103]	TAAAGCCTGGGGTGCCTACAGCTGGCGAAAGGGGGAACGGCGGAT
5[144]	3[151]	ACTGCCCGCTTTCCAGTCAGGCTGCGCAACTGTTGGAACCGTGCA
5[192]	3[199]	ATAAATCAATATATGTGATATTCCGGCACCGCTTCTCGCACTCCA
5[240]	3[247]	TTAATTTTCCCTTAGAATAAACGCAATAATAACGGAGGTGGCAAC
5[288]	3[295]	GAGAAGAGTCAATAGTGATTTTTAAGAAAAGTAAGCACAATCAAT
5[336]	3[343]	TAACCTCCGGCTTAGGTTCCAATAATAAGAGCAAGATTCAACCGA
5[384]	3[391]	CAATCGCAAGACAAAGAAGAGGGTAATTGAGCGCTATGAATTATC
5[432]	3[439]	TCATCTTCTGACCTAAATAAACAGGGAAGCGCATTAACCAGTAGC
5[480]	3[487]	GCGTTAAATAAGAATAAATTTTGTTTAACGTCAAAAGATAGCAGC
5[528]	3[535]	TATCATATGCGTTATACACAGTTACAAAATAAACAGCTGTAGCGC
5[576]	3[583]	GGCTTAATTGAGAATCGCTTTTATCCTGAATCTTACCATCTTTTC
5[624]	3[631]	GCATTTTCGAGCCAGTAAGGAGGTTTTGAAGCCTTACTCAGAGCC
5[672]	3[679]	TTCTGTCCAGACGACGACGCTTATCCGGTATTCTAAGCCACCAGA
5[720]	3[727]	GTTTATCAACAATAGATATTTCATCGTAGGAATCATGTCAGACGA
5[768]	3[775]	TACGAGCATGTAGAAACCAACGGGTATTAAACCAAGATGGAAAGC
6[791]	7[791]	CCAGCAGCACCGCCTGCA
7[8]	6[8]	GAGATAGGGATCCCTTAT
7[24]	7[55]	GTTCCAGTTTGGAACAAGAGTCCACTATTAAAGAAC
7[56]	2[48]	GTGGACTCCGGTTTGCCCCAGCAGGCGAATACTTTTGTAAACGTT
7[72]	7[103]	GGGCGAAAAACCGTCTATCATCGAGGTGCCGTAAAG
7[104]	2[96]	CACTAAATCCTGATTGCCCTTCACCGCCCCTCATATGATAATCAG
7[120]	7[151]	AAGGGAGCCCCCGATTTAGAGCTTGACGGGGAAAGC
7[152]	2[144]	CGGCGAACGTTGCGTATTGGGCGCCAGGGGTGAGAAGAATCGATG
7[168]	7[199]	AGGAAGGGAAGAAAGCGAAAGGAGCGGGCGCTAGGG
7[200]	2[192]	CGCTGGCAAATTTGAATTACCTTTTTTCTAGCTGATGAGAGATCT
7[216]	7[247]	TCACGCTGCGCGTAACCACCACACCCGCCGCGCTTA
7[248]	2[240]	ATGCGCCGCGAAGATGATGAAACAAACTAGTTGCGCAGGAGCCTT
7[264]	7[295]	CGTACTATGGTTGCTTTGACGAGCACGTATAACGTG
7[296]	2[288]	CTTTCCTCGCAAGTTACAAAATCGCGCTCGCTGAGGAATTGCGAA
7[312]	7[343]	GAGCGGGAGCTAAACAGGAGGCCGATTAAAGGGATT
7[344]	2[336]	TTAGACAGGGTACCTTTTACATCGGGACAGCGAAAGTAAACAACT
7[360]	7[391]	CCAGAATCCTGAGAAGTGTTTTTATAATCAGTGAGG
7[392]	2[384]	CCACCGAGTATAAAGAAATTGCGTAGACTAAAGACTAGTTTTGTC
7[408]	7[439]	TGTCCATCACGCAAATTAACCGTTGTAGCAATACTT
7[440]	2[432]	CTTTGATTAATAATGGAAGGGTTAGAAACTACGAAGTACAACGCC

7[456]	7[487]	TCACTTGCCTGAGTAGAAGAACTCAAACTATCGGCC
7[488]	2[480]	TTGCTGGTATCAGATGATGGCAATTCACATCTTTGAAAGCCCAAT
7[504]	7[535]	ACAATATTACCGCCAGCCATTGCAACAGGAAAAACG
7[536]	2[528]	CTCATGGAATGCGGAACAAAGAAACCATGTATCATCGCCACCCTC
7[552]	7[583]	TTTTGACGCTCAATCGTCTGAAATGGATTATTTACA
7[584]	2[576]	TTGGCAGATAAATCCTTTGCCCGAACGAGCCGGAACCGGAATAGG
7[600]	7[631]	ACACGACCAGTAATAAAAGGGACATTCTGGCCAACA
7[632]	2[624]	GAGATAGAAACATTTGAGGATTTAGAAGAAAGAGGATTGCTCAGT
7[648]	7[679]	CCTGAAAGCGTAAGAATACGTGGCACAGACAATATT
7[680]	2[672]	TTTGAATGGAAAATATCTTTAGGAGCAATCAAGAGTGAAAGTATT
7[696]	7[727]	TTTAATGCGCGAACTGATAGCCCTAAAACATCGCCA
7[728]	2[720]	TTAAAAATAAATATCTGGTCAGTTGGCAAGCTGCTCCGTATAAAC
7[744]	7[775]	CCACCAGCAGAAGATAAAACAGAGGTGAGGCGGTCA
7[776]	2[768]	GTATTAACAAATGAAAAATCTAAAGCAAGTAAATTGAGGAGTGTA

Table S3. Sequence of eight-helix DNA origami.

Name	Sequence
8h10b	/5Biosg/AAAGCCTCAGAGCATAAATTAGCAAAATTAAGCAAT
8h32b	/5Biosg/CAATAGGAACGCCATCAAATCAGCTCATTTTTTAAC
8h54b	/5Biosg/TCCCCGGGTACCGAGCTCCAGGTCGACTCTAGAGGA
8h76b	/5Biosg/GAATAGCCCGAGATAGGGATCCCTTATAAATCAAAA
8t23d	AGCGTCATACATGGCTTTGAATTTACCGTTCCAGTA/3Dig_N/
8t01d	AAT CAT TGT GAA TTA CCT TGG TTT AAT TTC AAC TTT /3Dig_N/
8t45d	TTT CCT TAT CAT TCC AAG AAT CAA TAA TCG GCT GTC /3Dig_N/
8t67d	CGCTGAGAGCCAGCAGCACCGCCTGCAACAGTGCCA /3Dig_N/
L8h1	CTTT AAAGCCTCAGAGCATAAATTAGCAAAATTAAG
L8h2	AGGAACGCCATCAA AAATAATTCGCGTCTGGCTACAGGCAA
L8h3	CGGGT ACCGAGCTC ATCAGCTCA TTTTT
L8h4	CAGGTCGAC TCTAGAGGA TTTC
L8h5	GCCA GAATAGCCCGAGATAGGGATCCCTTATAAATC
L8t1	CAAT AATCATTGTGAATTACCTTGGTTTAAT TTCAA
L8t2	CTTATCATTCCAAGAATCAATAA CATGGCTTT
L8t3	GCAGTCTCT GAATTTACCGTTCCAGTA CAAT
L8t4	TAAC AGCGTCATA TCGGCTGTC TCCC
L8t5	AAAA CGCTGAGAGCCAGCAGCACCGCCTGCAACAGT
PriF	/5Biosg/GAGTTTCCTGCTCCGTCTGACC
PriR	/5Dig_N/CATAGTGAAACGTATCCTCCCCG

Table S4. Sequence of biotin-, digoxigenin-labeled strands, linker strands and primers for dsDNA handles preparation.

CaE

Cluster ID	Area	Number of contacts	Contacts/Residue	Area/Residue
**0**	191.04	2	1	95.52
**1**	2892.49	65	2.71	44.5

NCS1

Cluster ID	Area	Number of contacts	Contacts/Residue	Area/Residue
**0**	637.63	13	2.17	49.05
**1**	91.31	4	1.33	22.83
**2**	756.87	16	2	47.3
**3**	173.98	4	1.33	43.49
**4**	166.89	4	1.33	41.72
**5**	52.3	2	1	26.15
**6**	52.3	2	1	26.15

CaM

Cluster ID	Area	Number of contacts	Contacts/Residue	Area/Residue
**0**	116.13	4	1.33	29.03
**1**	628.1	11	1.83	57.1
**2**	48.98	2	1	24.49
**3**	674.64	12	2	56.22
**4**	28.81	2	1	14.41

Table S5. Hydrophobic cluster analysis of proteins CaE, NCS1 and CaM (NMR data).

CaE

Network ID	Donor - Aceptor	Distance	Angle DHA (degrees)
**0**	ARG26-NH1 -- SER71-OG	2.92	133.31
**1**	ASN114-ND2 -- GLU123-OE2	3.09	160.95
**2**	THR76-OG1 -- GLN79-OE1	3.41	161.38
**2**	THR76-OG1 -- GLN79-NE2	2.79	137.94

NCS1

Network ID	Donor - Aceptor	Distance	Angle DHA (degrees)
**0**	ARG18-NE -- GLU14-OE2	2.35	121.64
**1**	ARG79-NE -- ASP44-OD1	2.36	126.34
**2**	ARG118-NH1 -- ASP157-OD2	3.21	129.3
**2**	ARG118-NH2 -- ASP157-OD2	2.6	166.75
**3**	TYR129-OH -- GLU142-OE2	2.49	179.45

CaM

Network ID	Donor - Aceptor	Distance	Angle DHA (degrees)
**0**	GLN135-NE2 -- ASP133-OD2	3.2	142.22
**1**	THR26-OG1 -- ASP24-OD1	3.27	163.76
**2**	THR29-OG1 -- GLU45-OE2	3.18	160.23
**3**	THR44-OG1 -- GLU47-OE1	3.26	164.73
**4**	TYR138-OH -- GLU82-OE2	2.35	160.8

Table S6. Hydrogen bond network analysis of proteins CaE, NCS1 and CaM (NMR data).

CaE

Network ID	Salt bridge	κ	Fraction of Charged Residues (FCR)
**0**	ASP-61 -- ARG-26	0.13	0.28
**1**	ARG-163 -- GLU-173		

NCS1

Network ID	Salt bridge	κ	Fraction of Charged Residues (FCR)
**0**	GLU-14 -- ARG-18	0.16	0.30
**1**	ASP-44 -- ARG-79		
**2**	ARG-118 -- ASP-157		

CaM

Network ID	Salt bridge	κ	Fraction of Charged Residues (FCR)
**0**	GLU-82 -- ARG-86	0.18	0.35

Table S7. Salt bridge network analysis of proteins CaE, NCS1 and CaM (NMR data).

CaE

Cluster ID	Area	Number of contacts	Contacts/Residue	Area/Residue
**0**	101.73	2	1	50.86
**1**	2755.08	76	2.92	36.25

NCS1

Cluster ID	Area	Number of contacts	Contacts/Residue	Area/Residue
**0**	746.45	21	2.62	35.55
**1**	161.57	4	1.33	40.39
**2**	564.05	17	1.89	33.18
**3**	524.38	10	2	52.44
**4**	64.27	2	1	32.14
**5**	66.27	2	1	33.13

CaM

Cluster ID	Area	Number of contacts	Contacts/Residue	Area/Residue
**0**	250	6	2	41.67
**1**	658.9	14	2.33	47.06
**2**	46.54	2	1	23.27
**3**	122.34	2	1	61.17
**4**	107.05	4	1.33	26.76
**5**	545.87	12	2	45.49

Table S8. Hydrophobic cluster analysis of proteins CaE, NCS1 and CaM (AlphaFold data).

CaE

Network ID	Donor - Aceptor	Distance	Angle DHA (degrees)
**0**	ARG9-NH2 -- ASP172-OD1	2.69	131.86
**1**	LYS11-NZ -- GLU78-OE1	2.75	155.38
**1**	LYS11-NZ -- GLU78-OE2	2.77	126.84
**2**	LYS12-NZ -- ASP8-OD2	2.72	129.98
**3**	ARG13-NE -- GLU174-OE1	3.1	141.46
**3**	ARG13-NH2 -- GLU174-OE1	2.9	150.7
**4**	TRP17-NE1 -- GLU33-OE1	3.15	122.51
**5**	ASN22-ND2 -- ASP20-OD2	2.9	120.91
**6**	ARG27-NH1 -- ASP62-OD2	3.01	167.97
**7**	GLN51-NE2 -- ASN55-ND2	3.08	144.89
**8**	GLN92-NE2 -- GLU67-OE2	3.02	122.26
**8**	GLN92-NE2 -- ASN87-OD1	3.24	165.58
**9**	ARG170-NH1 -- ASP165-OD1	2.96	155.66
**10**	SER7-OG -- GLU86-OE1	2.62	157.59
**11**	THR77-OG1 -- GLN80-OE1	2.78	173.98
**12**	SER135-OG -- GLU138-OE2	2.71	164.06
**13**	THR148-OG1 -- GLU158-OE1	3.04	165.21
**13**	SER155-OG -- GLU158-OE2	2.74	177.89

NCS1

Network ID	Donor - Aceptor	Distance	Angle DHA (degrees)
**0**	LYS19-NZ -- GLU15-OE1	2.66	120.81
**1**	GLN42-NE2 -- GLU81-OE1	2.98	155.8
**2**	GLN49-NE2 -- THR66-OG1	2.93	140.81
**3**	LYS63-NZ -- GLN130-NE2	3.14	137.61
**3**	LYS63-NZ -- ASP126-OD2	2.99	132.09
**4**	ASN75-ND2 -- ASP77-OD1	3.44	168.04
**5**	ARG94-NH1 -- GLU26-OE1	2.87	140.83
**5**	ARG94-NH1 -- GLU26-OE2	3.34	156.07
**5**	ARG94-NH2 -- VAL190-OXT	3.06	149.2
**5**	ARG94-NH2 -- GLU26-OE1	2.74	150.58
**6**	LYS100-NZ -- ASP187-OD1	2.53	149.69
**7**	ARG118-NH1 -- ASP150-OD1	2.66	165.49
**3**	GLN130-NE2 -- LYS63-NZ	3.14	152.04
**3**	GLN130-NE2 -- ASP126-OD1	2.9	122.84
**8**	LYS147-NZ -- ASN143-OD1	2.87	167.31
**9**	ARG148-NH1 -- GLU142-OE1	2.69	123.28
**10**	ASN159-ND2 -- ASP161-OD1	3.19	159.15
**11**	GLN167-NE2 -- GLU171-OE1	2.83	161.95
**12**	LYS174-NZ -- ASP98-OD1	2.61	142.6
**13**	TYR21-OH -- GLU99-OE2	2.98	163.88
**5**	THR23-OG1 -- GLU26-OE2	2.77	169.33
**14**	THR62-OG1 -- ASP60-OD1	2.56	160.24
**13**	THR96-OG1 -- GLU99-OE2	2.74	171.91
**15**	THR117-OG1 -- GLU120-OE2	2.71	168.74
**16**	THR144-OG1 -- GLU146-OE2	2.89	151.81
**17**	THR165-OG1 -- GLU168-OE2	2.74	174.4
**18**	SER178-OG -- ASP176-OD1	2.58	176.25

CaM

Network ID	Donor - Aceptor	Distance	Angle DHA (degrees)
**0**	GLN50-NE2 -- ASN54-OD1	2.92	161.08
**1**	ARG87-NH2 -- GLU83-OE1	2.62	141.16

**2**	ARG107-NH1 -- ASP119-OD1	2.95	135.98
**3**	ASN112-ND2 -- HIS108-NE2	3.1	147.68
**4**	THR27-OG1 -- ASP25-OD1	2.69	177.39
**5**	THR29-OG1 -- GLU32-OE2	2.77	174.02
**6**	THR30-OG1 -- GLU46-OE1	2.66	161.16
**7**	THR45-OG1 -- GLU48-OE2	2.79	168.44
**8**	THR63-OG1 -- ASN61-OD1	2.65	176.67
**9**	SER102-OG -- GLU105-OE2	2.69	174.57
**10**	THR118-OG1 -- GLU121-OE2	2.8	170.61

Table S9. Hydrogen bond network analysis of proteins CaE, NCS1 and CaM (AlphaFold data).

CaE

Network ID	Salt bridge	κ	Fraction of Charged Residues (FCR)
0	ARG-9 -- ASP-172	0.13	0.28
1	LYS-11 -- GLU-78		
2	ASP-8 -- LYS-12		
3	ARG-13 -- ARG-16 -- GLU-174		
4	ARG-27 -- ASP-62		
5	ARG-27 -- ASP-62		
6	ARG-27 -- ASP-62		

NCS1

Network ID	Salt bridge	κ	Fraction of Charged Residues (FCR)
0	LYS-19 -- GLU-15	0.16	0.30
1	LYS-63 -- ASP-126		
2	ARG-94 -- GLU-26		
3	ASP-187 -- LYS-100		
4	ARG-118 -- ASP-150		
5	ARG-151 -- GLU-142 -- ARG-148		
6	LYS-174 -- ASP-98		

CaM

Network ID	Salt bridge	κ	Fraction of Charged Residues (FCR)
0	GLU-83 -- ARG-87	0.18	0.35
1	ARG-107 -- ASP-119		

Table S10. Salt bridge network analysis of proteins CaE, NCS1 and CaM (AlphaFold data).

REFERENCES AND NOTES

1. F. U. Hartl, M. Hayer-Hartl, Converging concepts of protein folding in vitro and in vivo. *Nat. Struct. Mol. Biol.* **16**, 574–581 (2009).

2. D. H. Goldman, C. M. Kaiser, A. Milin, M. Righini, I. Tinoco Jr., C. Bustamante, Mechanical force releases nascent chain–mediated ribosome arrest in vitro and in vivo. *Science* **348**, 457–460 (2015).

3. K. Neupane, D. A. N. Foster, D. R. Dee, H. Yu, F. Wang, M. T. Woodside, Direct observation of transition paths during the folding of proteins and nucleic acids. *Science* **352**, 239–242 (2016).

4. H. Yu, M. G. W. Siewny, D. T. Edwards, A. W. Sanders, T. T. Perkins, Hidden dynamics in the unfolding of individual bacteriorhodopsin proteins. *Science* **355**, 945–950 (2017).

5. F. U. Hartl, Protein misfolding diseases. *Annu. Rev. Biochem.* **86**, 21–26 (2017).

6. J. Jumper, R. Evans, A. Pritzel, T. Green, M. Figurnov, O. Ronneberger, K. Tunyasuvunakool, R. Bates, A. Žídek, A. Potapenko, A. Bridgland, C. Meyer, S. A. A. Kohl, A. J. Ballard, A. Cowie, B. Romera-Paredes, S. Nikolov, R. Jain, J. Adler, T. Back, S. Petersen, D. Reiman, E. Clancy, M. Zielinski, M. Steinegger, M. Pacholska, T. Berghammer, S. Bodenstein, D. Silver, O. Vinyals, A. W. Senior, K. Kavukcuoglu, P. Kohli, D. Hassabis, Highly accurate protein structure prediction with AlphaFold. *Nature* **596**, 583–589 (2021).

7. J. Abramson, J. Adler, J. Dunger, R. Evans, T. Green, A. Pritzel, O. Ronneberger, L. Willmore, A. J. Ballard, J. Bambrick, S. W. Bodenstein, D. A. Evans, C.-C. Hung, M. O'Neill, D. Reiman, K. Tunyasuvunakool, Z. Wu, A. Žemgulytė, E. Arvaniti, C. Beattie, O. Bertolli, A. Bridgland, A. Cherepanov, M. Congreve, A. I. Cowen-Rivers, A. Cowie, M. Figurnov, F. B. Fuchs, H. Gladman, R. Jain, Y. A. Khan, C. M. R. Low, K. Perlin, A. Potapenko, P. Savy, S. Singh, A. Stecula, A. Thillaisundaram, C. Tong, S. Yakneen, E. D. Zhong, M. Zielinski, A. Žídek, V. Bapst, P. Kohli, M. Jaderberg, D. Hassabis, J. M. Jumper, Accurate structure prediction of biomolecular interactions with AlphaFold 3. *Nature* **630**, 493–500 (2024).

8. F. Capozzi, F. Casadei, C. Luchinat, EF-hand protein dynamics and evolution of calcium signal transduction: An NMR view. *J. Biol. Inorg. Chem.* **11**, 949–962 (2006).

9. Z. Grabarek, Structural basis for diversity of the EF-hand calcium-binding proteins. *J. Mol. Biol.* **359**, 509–525 (2006).

10. S. Mukherjee, P. M. K. Mohan, K. Kuchroo, K. V. R. Chary, Energetics of the native energy landscape of a two-domain calcium sensor protein: Distinct folding features of the two domains. *Biochemistry* **46**, 9911–9919 (2007).

11. J. Stigler, F. Ziegler, A. Gieseke, J. C. M. Gebhardt, M. Rief, The complex folding network of single calmodulin molecules. *Science* **334**, 512–516 (2011).

12. P. O. Heidarsson, M. R. Otazo, L. Bellucci, A. Mossa, A. Imparato, E. Paci, S. Corni, R. Di Felice, B. B. Kragelund, C. Cecconi, Single-molecule folding mechanism of an EF-hand neuronal calcium sensor. *Structure* **21**, 1812–1821 (2013).

13. P. O. Heidarsson, M. M. Naqvi, M. R. Otazo, A. Mossa, B. B. Kragelund, C. Cecconi, Direct single-molecule observation of calcium-dependent misfolding in human neuronal calcium sensor-1. *Proc. Natl. Acad. Sci. U.S.A.* **111**, 13069–13074 (2014).

14. D. G. Swan, R. S. Hale, N. Dhillon, P. F. Leadlay, A bacterial calcium-binding protein homologous to calmodulin. *Nature* **329**, 84–85 (1987).

15. H. Aitio, T. Laakso, T. Pihlajamaa, M. Torkkeli, I. Kilpeläinen, T. Drakenberg, R. Serimaa, A. Annila, Characterization of apo and partially saturated states of calerythrin, an EF-hand protein from S. *erythraea*: A molten globule when deprived of Ca^{2+}. *Protein Sci.* **10**, 74–82 (2001).

16. H. Tossavainen, P. Permi, A. Annila, I. Kilpeläinen, T. Drakenberg, NMR solution structure of calerythrin, an EF-hand calcium-binding protein from Saccharopolyspora erythraea. *Eur. J. Biochem.* **270**, 2505–2512 (2003).

17. C. Cecconi, E. A. Shank, C. Bustamante, S. Marqusee, Direct observation of the three-state folding of a single protein molecule. *Science* **309**, 2057–2060 (2005).

18. C. M. Kaiser, D. H. Goldman, J. D. Chodera, I. Tinoco, C. Bustamante, The ribosome modulates nascent protein folding. *Science* **334**, 1723–1727 (2011).

19. L. M. Alexander, D. H. Goldman, L. M. Wee, C. Bustamante, Non-equilibrium dynamics of a nascent polypeptide during translation suppress its misfolding. *Nat. Commun.* **10**, 2709 (2019).

20. C. Cecconi, E. A. Shank, F. W. Dahlquist, S. Marqusee, C. Bustamante, Protein-DNA chimeras for single molecule mechanical folding studies with the optical tweezers. *Eur. Biophys. J.* **37**, 729–738 (2008).

21. C. Bustamante, L. Alexander, K. Maciuba, C. M. Kaiser, Single-molecule studies of protein folding with optical tweezers. *Annu. Rev. Biochem.* **89**, 443–470 (2020).

22. N. Forns, S. de Lorenzo, M. Manosas, K. Hayashi, J. M. Huguet, F. Ritort, Improving signal/noise resolution in single-molecule experiments using molecular constructs with short handles. *Biophys. J.* **100**, 1765–1774 (2011).

23. E. Pfitzner, C. Wachauf, F. Kilchherr, B. Pelz, W. M. Shih, M. Rief, H. Dietz, Rigid DNA beams for high-resolution single-molecule mechanics. *Angew. Chem. Int. Ed. Engl.* **52**, 7766–7771 (2013).

24. F. Kilchherr, C. Wachauf, B. Pelz, M. Rief, M. Zacharias, H. Dietz, Single-molecule dissection of stacking forces in DNA. *Science* **353**, aaf5508 (2016).

25. H. K. Wayment-Steele, A. Ojoawo, R. Otten, J. M. Apitz, W. Pitsawong, M. Homberger, S. Ovchinnikov, L. Colwell, D. Kern, Predicting multiple conformations via sequence clustering and AlphaFold2. *Nature* **625**, 832–839 (2024).

26. Y. Ke, N. V. Voigt, K. V. Gothelf, W. M. Shih, Multilayer DNA origami packed on hexagonal and hybrid lattices. *J. Am. Chem. Soc.* **134**, 1770–1774 (2012).

27. C. Bustamante, J. Marko, E. Siggia, S. Smith, Entropic elasticity of lambda-phage DNA. *Science* **265**, 1599–1600 (1994).

28. R. D. Moore, G. A. Morrill, A possible mechanism for concentrating sodium and potassium in the cell nucleus. *Biophys. J.* **16**, 527–533 (1976).

29. M. Varadi, S. Anyango, M. Deshpande, S. Nair, C. Natassia, G. Yordanova, D. Yuan, O. Stroe, G. Wood, A. Laydon, A. Žídek, T. Green, K. Tunyasuvunakool, S. Petersen, J. Jumper, E. Clancy, R. Green, A. Vora, M. Lutfi, M. Figurnov, A. Cowie, N. Hobbs, P. Kohli, G. Kleywegt, E. Birney, D. Hassabis, S. Velankar, AlphaFold Protein Structure Database: Massively expanding the structural coverage of protein-sequence space with high-accuracy models. *Nucleic Acids Res.* **50**, D439–D444 (2022).

30. S. Sudhakar, M. K. Abdosamadi, T. J. Jachowski, M. Bugiel, A. Jannasch, E. Schäffer, Germanium nanospheres for ultraresolution picotensiometry of kinesin motors. *Science* **371**, eabd9944 (2021).

31. X. Shan, F. Wang, D. Wang, S. Wen, C. Chen, X. Di, P. Nie, J. Liao, Y. Liu, L. Ding, P. J. Reece, D. Jin, Optical tweezers beyond refractive index mismatch using highly doped upconversion nanoparticles. *Nat. Nanotechnol.* **16**, 531–537 (2021).

32. S. M. Douglas, A. H. Marblestone, S. Teerapittayanon, A. Vazquez, G. M. Church, W. M. Shih, Rapid prototyping of 3D DNA-origami shapes with caDNAno. *Nucleic Acids Res.* **37**, 5001–5006 (2009).

33. J. R. Moffitt, Y. R. Chemla, D. Izhaky, C. Bustamante, Differential detection of dual traps improves the spatial resolution of optical tweezers. *Proc. Natl. Acad. Sci. U.S.A.* **103**, 9006–9011 (2006).

34. P. J. Elms, J. D. Chodera, C. J. Bustamante, S. Marqusee, Limitations of constant-force-feedback experiments. *Biophys. J.* **103**, 1490–1499 (2012).

35. C. P. Robert, G. Celeux, J. Diebolt, Bayesian estimation of hidden Markov chains: A stochastic implementation. *Stat. Probab. Lett.* **16**, 77–83 (1993).

36. R. W. Katz, On some criteria for estimating the order of a Markov chain. *Dent. Tech.* **23**, 243–249 (1981).

37. J. Stigler, M. Rief, Hidden Markov analysis of trajectories in single-molecule experiments and the effects of missed events. *ChemPhysChem* **13**, 1079–1086 (2012).

38. R. K. Das, R. V. Pappu, Conformations of intrinsically disordered proteins are influenced by linear sequence distributions of oppositely charged residues. *Proc. Natl. Acad. Sci. U.S.A.* **110**, 13392–13397 (2013).

39. M. Mirdita, K. Schütze, Y. Moriwaki, L. Heo, S. Ovchinnikov, M. Steinegger, ColabFold: Making protein folding accessible to all. *Nat. Methods* **19**, 679–682 (2022).

40. M. Steinegger, J. Söding, MMseqs2 enables sensitive protein sequence searching for the analysis of massive data sets. *Nat. Biotechnol.* **35**, 1026–1028 (2017).

41. E. C. Meng, T. D. Goddard, E. F. Pettersen, G. S. Couch, Z. J. Pearson, J. H. Morris, T. E. Ferrin, U. C. S. F. ChimeraX, Tools for structure building and analysis. *Protein Sci.* **32**, e4792 (2023).

42. R. T. McGibbon, K. A. Beauchamp, M. P. Harrigan, C. Klein, J. M. Swails, C. X. Hernández, C. R. Schwantes, L.-P. Wang, T. J. Lane, V. S. Pande, MDTraj: A modern open library for the analysis of molecular dynamics trajectories. *Biophys. J.* **109**, 1528–1532 (2015).

43. F. Pedregosa, G. Varoquaux, A. Gramfort, V. Michel, B. Thirion, O. Grisel, M. Blondel, P. Prettenhofer, R. Weiss, V. Dubourg, Scikit-learn: Machine learning in Python. *J. Mach. Learn. Res.* **12**, 2825–2830 (2011).

44. T. R. Alderson, J. H. Lee, C. Charlier, J. Ying, A. Bax, Propensity for cis-proline formation in unfolded proteins. *Chembiochem* **19**, 37–42 (2018).

45. S. K. Sarkar, P. E. Young, C. E. Sullivan, D. A. Torchia, Detection of cis and trans X-Pro peptide bonds in proteins by 13C NMR: Application to collagen. *Proc. Natl. Acad. Sci. U.S.A.* **81**, 4800–4803 (1984).

46. J. A. Cox, I. Durussel, D. J. Scott, M. W. Berchtold, Remodeling of the AB site of rat parvalbumin and oncomodulin into a canonical EF-hand. *Eur. J. Biochem.* **264**, 790–799 (1999).

47. E. Babini, I. Bertini, F. Capozzi, C. Del Bianco, D. Hollender, T. Kiss, C. Luchinat, A. Quattrone, Solution structure of human β-parvalbumin and structural comparison with

its paralog α-parvalbumin and with their rat orthologs. *Biochemistry* **43**, 16076–16085 (2004).
